# Multifunctional Derivatives of Spiropyrrolidine Tethered Indeno-Quinoxaline Heterocyclic Hybrids as Potent Antimicrobial, Antioxidant and Antidiabetic Agents: Design, Synthesis, In Vitro and In Silico Approaches

**DOI:** 10.3390/molecules27217248

**Published:** 2022-10-25

**Authors:** Nouha Bouali, Manel Ben Hammouda, Iqrar Ahmad, Siwar Ghannay, Amira Thouri, Amal Dbeibia, Harun Patel, Walid Sabri Hamadou, Karim Hosni, Mejdi Snoussi, Mohd Adnan, Md Imtaiyaz Hassan, Emira Noumi, Kaïss Aouadi, Adel Kadri

**Affiliations:** 1 Department of Biology, College of Science, Hail University, P.O. Box 2440, Hail 2440, Saudi Arabia; 2 Laboratory of Heterocyclic Chemistry Natural Product and Reactivity/CHPNR, Department of Chemistry, Faculty of Science of Monastir, University of Monastir, Monastir 5000, Tunisia; 3 Division of Computer Aided Drug Design, Department of Pharmaceutical Chemistry, R. C. Patel Institute of Pharmaceutical Education and Research, Shirpur 425405, Maharashtra, India; 4 Department of Chemistry, College of Science, Qassim University, Buraidah 51452, Saudi Arabia; 5 Laboratory of Bioresources, Biology Integrative and Valorization, Higher Institute of Biotechnology of Monastir, University of Monastir, Monastir 5000, Tunisia; 6 Laboratory of Analyzes, Treatment and Valorization of Environmental Pollutants and Products, Faculty of Pharmacy of Monastir, University of Monastir, Monastir 5019, Tunisia; 7 Laboratoire des Substances Naturelles, Institute National de Recherche et d’Analyse Physico-Chimique, Biotechpôle de Sidi Thabet, Sidi Thabet 2020, Tunisia; 8 Laboratory of Genetics, Biodiversity and Valorization of Bio-Resources (LR11ES41), Higher Institute of Biotechnology of Monastir, University of Monastir, Avenue Tahar Haddad, BP74, Monastir 5000, Tunisia; 9 Center for Interdisciplinary Research in Basic Sciences, Jamia Millia Islamia, New Delhi 110025, India; 10 Department of Chemistry, Faculty of Science of Monastir, University of Monastir, Avenue of the Environment, Monastir 5019, Tunisia; 11 Department of Chemistry, Faculty of Science and Arts of Baljurashi, Albaha University, Al Bahah P.O. Box 1988, Saudi Arabia; 12 Department of Chemistry, Faculty of Science of Sfax, University of Sfax, B.P. 1171, Sfax 3000, Tunisia

**Keywords:** spiropyrrolidine derivatives, antimicrobial, antioxidant, antidiabetic, ADME, molecular docking and dynamic simulation

## Abstract

To combat emerging antimicrobial-resistant microbes, there is an urgent need to develop new antimicrobials with better therapeutic profiles. For this, a series of 13 new spiropyrrolidine derivatives were designed, synthesized, characterized and evaluated for their in vitro antimicrobial, antioxidant and antidiabetic potential. Antimicrobial results revealed that the designed compounds displayed good activity against clinical isolated strains, with **5d** being the most potent (MIC 3.95 mM against *Staphylococcus aureus* ATCC 25923) compared to tetracycline (MIC 576.01 mM). The antioxidant activity was assessed by trapping DPPH, ABTS and FRAP assays. The results suggest remarkable antioxidant potential of all synthesized compounds, particularly **5c**, exhibiting the strongest activity with IC_50_ of 3.26 ± 0.32 mM (DPPH), 7.03 ± 0.07 mM (ABTS) and 3.69 ± 0.72 mM (FRAP). Tested for their *α*-amylase inhibitory effect, the examined analogues display a variable degree of *α*-amylase activity with IC_50_ ranging between 0.55 ± 0.38 mM and 2.19 ± 0.23 mM compared to acarbose (IC_50_ 1.19 ± 0.02 mM), with the most active compounds being **5d**, followed by **5c** and **5j**, affording IC_50_ of 0.55 ± 0.38 mM, 0.92 ± 0.10 mM, and 0.95 ± 0.14 mM, respectively. Preliminary structure–activity relationships revealed the importance of such substituents in enhancing the activity. Furthermore, the ADME screening test was applied to optimize the physicochemical properties and determine their drug-like characteristics. Binding interactions and stability between ligands and active residues of the investigated enzymes were confirmed through molecular docking and dynamic simulation study. These findings provided guidance for further developing leading new spiropyrrolidine scaffolds with improved dual antimicrobial and antidiabetic activities.

## 1. Introduction

The emergence of multidrug-resistant bacteria, often called “superbugs”, has become a global public health issue according to the World Health Organization (WHO) [1,2]. The WHO has declared antimicrobial resistance to be one of the 10th greatest public health threats facing humanity [2]. The lack of new drug development as well as the misuse and overuse of antimicrobials remains the main factors that have led to the rise and spread of drug-resistant pathogens, which has slowed down the eradication process of infectious diseases [3]. Antibiotic resistance has gradually developed and now affects all pathogenic bacteria. It results from the repeated administration of antibiotics in humans or animals, which creates conditions, called “selection pressure” favoring the acquisition and dissemination of antibiotic-resistant strains [4]. Therefore, antibiotics and other antimicrobial drugs lose their effectiveness and infections become increasingly difficult or even impossible to treat and lead to death [5].

Diabetes mellitus (DM) is a noncommunicable disease (NCDs) that includes a group of metabolic conditions characterized by hyperglycemia and resulting from defects in insulin action, insulin secretion or both [6,7]. Chronic hyperglycemia is associated with considerable morbidity and mortality, especially from cardiovascular and renal failure, with long-term damage and dysfunction of the eyes, kidneys, blood vessels, nerves and heart [8,9,10]. It is furthermore a common belief that people with diabetes are generally more vulnerable to infectious disease than those without diabetes [11]. *α*-Amylase attracts more attention due to its potential ability on attacking of *α*-1,4-glycosidic linkage by hydrolysis and was known as a hydrolyze enzyme containing Ca^+2^ ion in its active pocket. Its main role is to catalyze the conversion of starch into glucose and maltose by involving water molecules [12,13]. Additionally, some medications such as acarbose, voglibose and miglitol, despite their adverse side effects, have been employed as inhibitors of the hydrolyzing ability of the *α*-amylase enzyme and consequently blocked the absorption of glucose [14]. 

**Figure 1:**
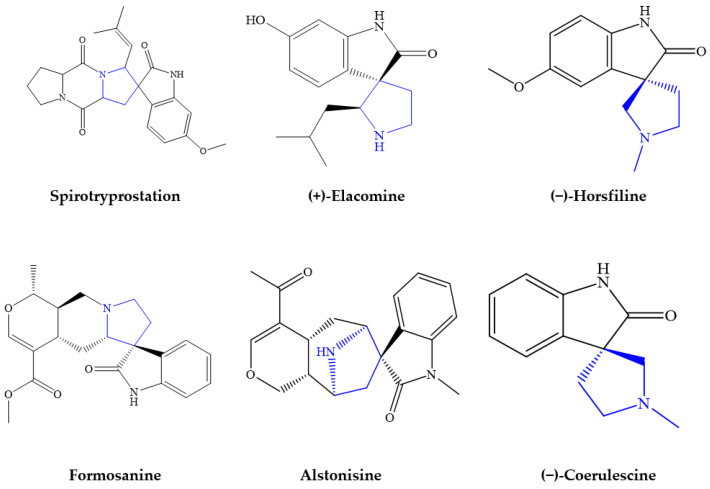
Highly pronounced biological spiropyrrolidine derivatives.

Quinoxalines, also known as benzo[a]pyrazines, are nitrogen-containing heterocycles scaffolds that have attracted enormous attention in synthetic and medicinal chemistry because of their widespread prevalence in nature. In addition, indeno [1,2-b]quinoxaline, as a privileged core with active pharmacophore and therapeutic value such as anticancer [15], antiproliferative [16] and Jun N-terminal kinase inhibitory effects [17], was used in the elaboration of various spiro-polycyclic frameworks. In this perspective, spiropyrrolidine derivatives play an important role in both medicinal chemistry and drug discovery, owing to their prominent pharmaceutical potency, including antileaucamic, anticonvulsant antiviral and local anesthetic activities [18]. They also exhibited antimicrobial [19], antibacterial [20], antifungal [20], antimalarial [20], anti-inflammatory [21], analgesic [21], antidiabetic [22], antitubercular [23], antiproliferative [24], anticholinesterase [25], anticancer [26], and antimycobacterial [27] properties. Spirotryprostatin, (+)-elacomine, (−)-horsfiline, formosanine, alstonisine and (−)-coerulescine (Figure 1) are among the most relevant spiropyrrolidine analogues.

Moreover, the spirocyclic moieties, especially those with pyrrolidine-embedded spiroatom are pharmacologically very attractive because of their interesting therapeutic activities, such antimicrobial [28], antiproliferative [29] and anti-acetylcholinesterase (AChE) and antibutylcholinesterase (BChE) activities [30]. Motivated by the aforementioned information and in connection to our previous studies in developing antimicrobial [31,32,33,34,35,36,37,38,39,40,41,42], antioxidant [39,40,41,42,43,44,45,46,47,48] and antidiabetic [7,8,10] agents, we herein disclose the design, synthesis and biological evaluation of a series of a novel class of spiropyrrolidine tethered indeno-quinoxaline heterocyclic hybrids. To determine the role of different functionalities in the formation of a ligand–protein complex, an in silico molecular docking study of the most potent derivatives was performed against *S. aureus* tyrosyl-tRNA synthetase (PDB ID, 1JIJ), human peroxiredoxin 5 (PRDX5) (PDB code: 1HD2) and human pancreatic *α*-amylase (PDB code: 2QV4), followed by an ADMET study.

## 2. Results and Discussion

### 2.1. Chemistry

**Scheme 1:**
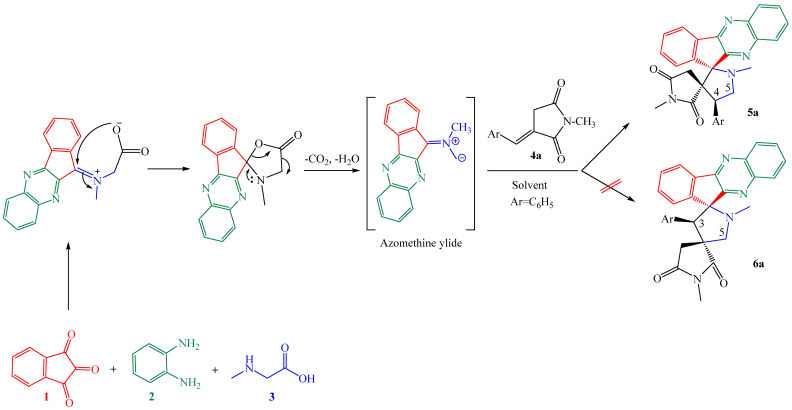
1, 3-Dipolar cycloaddition leading spiro[indenoquinoxaline-pyrrolidines] **5a**.

As shown in Scheme 1, we have chosen as a model the following four components: ninhydrin 1, o-phenylenediamine 2, sarcosine 3 and (*E*)-3-arylidene-1-methylpyrrolidine-2,5-dione 4a (Ar = C6H5) [49,50]. The reaction was carried out at different temperatures in acetonitrile and methanol. The obtained results show that the regio- and the stereoselectivity of this reaction depend both on the solvent and on the temperature (Table 1). Indeed, this optimization study revealed that the best results were obtained by refluxing the reaction mixture in methanol for 4 h, providing spiro[indenoquinoxaline-pyrrolidines] 5a with an excellent yield (81%) (Table 1, entry 5). Increasing the reaction time has no effect on the reaction yield.

**Scheme 2:**
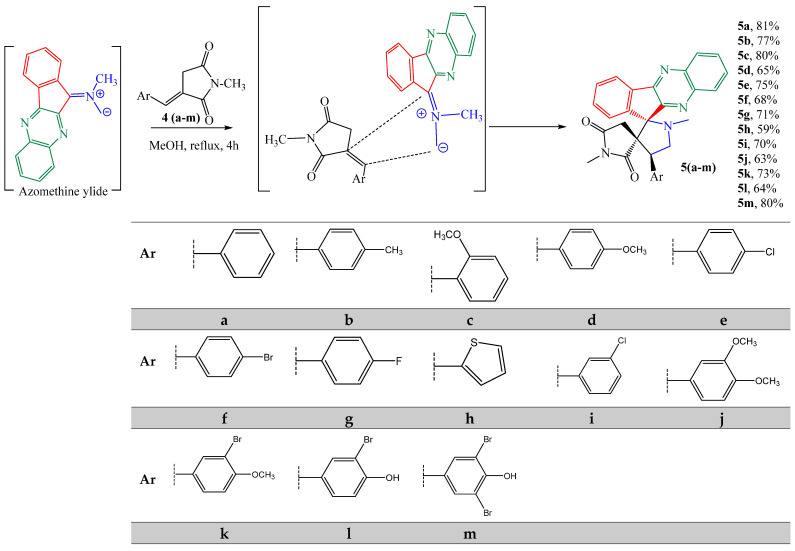
Substrate scope. All reactions were carried out with **1** (0.5 mmol), **2** (0.5 mmol), **3** (0.75 mmol) and **4a–m** (0.5 mmol) in MeOH (5.0 mL) at reflux for 4 h. Yields of the isolated cycloadducts are given.

After establishing the appropriate reaction conditions (Table 1, entry **5**), we tried to extend the scope of this reaction by using a series of different dipolarophiles, **4a–m**. This was performed in order to examine the influence of the electronic effects exerted by the substituent in positions *o*, *m* and *p* of the aryl group of imides **4** on the result of the reaction (Scheme 2). Both electron donating and electron-withdrawing groups exerted by the substituent at the o, m or *p*-position of the aryl group of imides **4** have a very limited influence on the efficiency of the cycloaddition reaction. For example, dipolarophiles 4 bearing electron-neutral (H), or electron-withdrawing substituent (e.g., *p*-Cl, *m*-Cl), or electron-donating (e.g., *p*-OMe or *m*-OMe) groups reacted smoothly to give spiro[indenoquinoxaline-pyrrolidines] products **5a–m** in good yields along with excellent diastereoselectivities (Scheme 2).

The structures and the relative configuration of the isolated spiropyrrolidines **5a–m** were elucidated by analyzing their spectroscopic data (NMR1D and NMR 2D).

### 2.2. Spectroscopic of the Isomeric Cycloadducts

**Figure 2:**
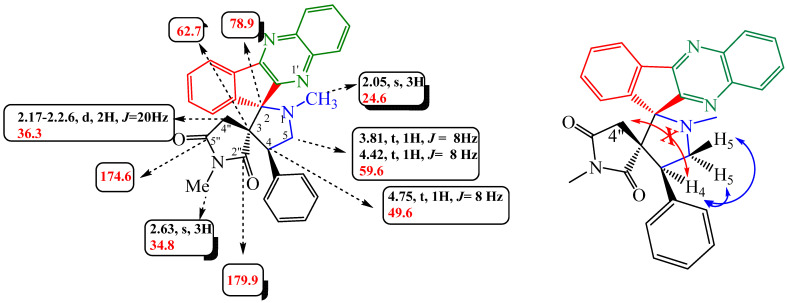
Selected ^1^H and ^13^C NMR (red) chemical shifts (ppm) (**left**) and the NOESY correlations (**right**) of **5a**.

The specific regioisomer was determined on the basis of the ^1^H NMR chemical shifts of the H-4 and H-5 protons [51] (Figure 2). 

The ^1^H NMR spectrum of **5a** shows the –NCH3 proton of the pyrrolidine ring exhibited a singlet at 2.05 ppm. Two mutually coupled doublets at δ 2.17–2.26 ppm were observed with a 2J coupling of 20 Hz, corresponding to the 4″-CH_2_ group. At 2.63 ppm, a three-proton integration singlet attributable to the Me–N protons of the pyrrolidine-2,5-dione ring. The H-4 and 5-CH_2_ protons appear as three triplets at 3.81, 4.42 and 4.75 ppm (*J* = 8 Hz), respectively (Figure 2). The multiplicity of the signals, three triplets, are clearly corroborating the regiochemistry of the cycloaddition reaction. If the hypothetical alternative regioisomer **6a** (Scheme 1) would have been formed, the pyrrolidinyl protons H-3 and 5-CH_2_ should give rise to a singlet and two doublet patterns in the ^1^H NMR spectrum. The aromatic protons found in the region between δ 7.31 and 8.27 ppm appear as a multiplet. The ^13^C NMR spectrum shows two peaks at δ 62.7 and 78.9 ppm, corresponding to the two spirocarbons C-3 and C-2. The pyrrolizidinyl carbons C-4 and C-5 appear at δ 49.6 and 59.6 ppm, respectively. In addition, the resonances at δ 174.6 ppm and δ 179.9 ppm are due to groups of pyrrolidine-2,5-dione. Experimental 2D NMR methods such as the NOESY, COSY and HMBC were used to further determine the stereo- and regioselectivity of the 1,3-dipolar cycloaddition leading to the **5a–m** cycloadducts. The HMBC spectrum of **5a** (see supplementary materials) indicated the following significant correlation between H4/C4”; H5/C4; H4/C5; H4/C3; CH2/C3 and H5/C3. The NOESY spectrum of compounds **5a** (see supplementary materials) shows correlations between H4/H5 protons; H4/HAr; H5/HAr, H5/N-CH_3_ (pyrrolidine) and H4”/H5 protons. Additionally, no correlation was observed between H4 and H4” protons. The COSY spectrum of **5a** (see supplementary materials) shows correlations between H4/H5 protons. The ʋ(C=O) absorptions at 1695 cm^−1^ in the IR spectrum of **5a** are due to the carbonyl groups of the imide. All these observations further confirm the stereochemistry proposed in (Figure 2). 

It is worth noting that in all cases, the reactions were found to be highly regioselective, leading to the generation of only one regioisomer **5**. No indication for the copresence of isomer **6**, even in smaller amounts, was found (Scheme 1).

### 2.3. Biological Screening

#### 2.3.1. Antimicrobial Activity vs. Structure Activity Relationship Studies

The antimicrobial screening of the synthesized compounds **5a–m** was investigated using the disc diffusion method towards different pathogenic strains. The results depicted in Table 1 are expressed quantitatively by MIC, MBC, MFC, MBC/MIC and MFC/MIC values. As shown, all compounds displayed potent antibacterial and moderate antifungal activities against the tested strains when compared to the standard drug, tetracycline and amphotericin B, respectively. A structure–activity relationship study confirmed that relocation of same substituent on phenyl ring affects the biological activity of the synthesized derivatives. Among the obtained series, compound **5d**, with a methoxy group attached to the phenyl ring at the *p*-position, displayed the highest antibacterial activity compared to the standard tetracycline against most of the tested bacterial strains. Nevertheless, the presence of the methoxy group at the *o*-position (**5c**) seems to decrease the antimicrobial activity. This decreased was less pronounced in **5j**, having another methoxy group in the *m*-position when compared to **5d**. Replacing the *m*-methoxy group (**5j**) with a bromo group (**5k**) decreased the antibacterial activity, especially against *S. aureus* but did not affect the antifungal potency. Additionally, compounds **5e** with a chloro group and **5g** with a fluoro group attached to the *p*-position of the phenyl ring showed equipotent activity; however, when replaced by the bulky bromo groups at the *p*-position (**5f**), a decrease in the antimicrobial activity was detected, and overall, all the strains may be explained by the bulkiness of this group during its binding mode to the cell of the microbes via the activating the apoptosis processes. Further, compounds **5i** with the chloro group at the *m*-position of the phenyl ring showed reduced antibacterial activity and similar antifungal activity when compared to **5e** (chloro group at *p*-position). Moreover, **5i**, with a hydroxy group at the *p*-position and the bromo group at the *m*-position of the phenyl ring showed weaker activity than **5m** substituted with another bulky bromo group on the second *m*-position. 

The analysis of the bactericidal (MBC/MIC ≤ 4)/bacteriostatic (MBC/MIC > 4) or fungicidal (MFC/MIC ≤ 4)/fungistatic (MFC/MIC > 4) characteristics of our compounds revealed that, except against *S. aureus* for **5c–e**, **5g–h**, and **5j–i**, the remaining compounds exhibited bactericidal and fungicidal characteristics towards all tested strains (Table 2).

#### 2.3.2. Antioxidant Activity vs. Structure Activity Relationship Studies

Free radicals are well-known to play a pivotal role in the pathogenesis of various human diseases causing damage to cells. Thus, it is of utmost importance to protect us against free radicals and save our health. Indeed, to assess their versatile synthetic applicability, the antioxidant potency of our synthesized new compounds was assessed using DPPH, ABTS and FRAP assays and compared to that of Trolox as a standard. The results were expressed by their IC_50_ values, the effective concentration at which 50% of the radicals were scavenged (Table 3). 

Antioxidant compounds donate a hydrogen atom or electrons to DPPH and convert it to a stable molecule, 1,1-diphenyl-picryl hydrazine. Following the DPPH radical scavenging test, compounds **5c** with a methoxy group at the o-position, **5d** with a methoxy group at the m-position, **5e** with a chloro group at the *p*-position and **5i** with a hydroxy group at the *p*-position showed potent antioxidant activity with IC_50_ values of 3.26 ± 0.32 mM, 6.12 ± 0.01 mM, 7.44 ± 0.15 mM and 7.80 ± 0.32 mM, respectively. Interestingly, **5a** with unsubstituted benzene, **5f** with a bromo group at the *m*-position and **5i** with a hydroxy group at the *p*-position and bromo group at the *m*-position of the phenyl ring displayed equipotent DPPH scavenging activity; however, incorporating a methoxy group at the para-position of the phenyl ring (**5b**) reduced the activity two-times, and the remaining compounds **5m** and **5k** displayed the weakest activity, exceeding the standard Trolox. Further, compounds **5e** with a chloro group (IC_50_ = 6.12 ± 0.01), **5f** with a bromo group (IC_50_ = 16.13 ± 0.39) and **5g** with a fluoro group (IC_50_ = 26.03 ± 0.50) attached to the *p*-position of the phenyl ring showed excellent scavenging DPPH effects due to the electron-withdrawing inductive effect of halogens, with **5e** exerting a 5.1-fold better DPPH scavenging activity than the standard, Trolox. Nonetheless, the presence of a chloro group at the *m*-position of the aryl ring decreases the DPPH scavenging activity due to the loss of electron density. Remarkably, **5k**, bearing p-methoxy and *m*-bromo groups, turned out to the less active compound due to the presence of bulky bromine group at the *m*-position. 

The antioxidant results related to the capture of ABTS radical revealed that out of the synthesized analogues, **5c**, **5g** and **5k** exhibited the strongest scavenging activity. An enhancement in the activity has been shown when replacing the m-methoxy group (**5j**) with a bromo group (**5k**). On the other hand, compounds 5g, bearing a fluoro group, displayed better ABTS scavenging activity than **5e** with a chloro group and **5f** with the bulky bromo group at the p-aromatic position, which remain about 14, 8 and 6-fold higher than Trolox, respectively. 

Based on the FRAP test, our results indicate that, except **5b**, all compounds showed more potent antioxidant activity than the standard, Trolox, with **5c** bearing methoxy group at *o*-position of the phenyl ring has the greatest antioxidant activity, followed by **5i**, **5h** and **5e** exhibiting similar activity, about 6.5-fold higher than Trolox. Although compounds **5e** with a chloro group displayed higher activity than **5g** with a fluoro group attached to the *p*-position of the phenyl ring, when replaced by the bulky bromo groups at the *p*-position (**5f**), the activity remains much higher.

#### 2.3.3. Antidiabetic Activity vs. Structure Activity Relationship Studies

The inhibitory effects of the synthesized analogues against *α*-amylase have been assessed as a strategy in lowering the postprandial hyperglycemia. The highest inhibitory effect was recorded to compound **5d** (0.55 ± 0.38 mM) with a methoxy group at the *p*-position, followed by **5c** (0.92 ± 0.10 mM) with a methoxy group at the *o*-position and **5j** (0.95 ± 0.14 mM) with two methoxy groups at the *p*- and *m*-positions exceeding that of acarbose (1.19 ± 0.02 mM). The decreased activity from **5d** to **5j**, which differs only by the presence of the m-methoxy group, was related to its electron-withdrawing effect. In addition to that, **5a** displayed similar activity to acarbose and the remaining compounds have shown lower activity. The presence of a methyl group with electron-donating effect also decreased the activity when compared to **5a**. The presence of different halogen groups (**5e**, **5f** and **5g**) in the *p*-position of the phenyl ring does not have a significant influence on the activity. The remaining analogues also displayed potent *α*-amylase inhibition (Table 3).

Based on the aforementioned SAR analysis, we assume that electron-withdrawing substituents increase the activity by increasing the polarity within/around the molecules, respectively.

### 2.4. Computational Studies

#### Druglikeness and Pharmacokinetics

The SwissADME web-based server was employed to predict ADME (absorption, distribution, metabolism, excretion) properties that cover the pharmacokinetic issues and determine whether a drug molecule reaches the target protein in the body and how long it stays in the bloodstream. They also provide a baseline for further in vivo trials and are considered as major steps for drugs targeting the central nervous system (CNS) because the ability of CNS drugs to penetrate the blood–brain barrier is of prime importance in drug metabolism. All compounds meet the Lipinski’s rule of five that states that an orally active drug has the number of hydrogen bond acceptors (HBA) ≤ 10, hydrogen bond donors (HBD) ≤ 5, molecular weight (MW) < 500 Da and Log P (the logarithm of octanol water partition coefficient) ≤ 5 and possesses a good bioavailability score of 0.55. They exhibited a lipophilicity given by the consensus Log Po/w in the range of 2.73–3.29. They displayed high GI (gastrointestinal) absorption, with only 5e and 5i were found to be BBB (blood-brain barrier) permeant, and **5c**, **5d** and **5j** were P-gp substrates. Cytochrome P450s, known as an important enzyme system for drug metabolism in the liver with CYP2D6 and CYP3A4, which are the two main subtypes of cytochrome P450, with the latter responsible for the metabolism of about 60% of xenobiotics, including drugs, carcinogens, steroids, and eicosanoids. The predicted results revealed that the selected compound was CYP2D6, but all were found to be CYP3A4 inhibitors. The skin permeability (log Kp) defines the rate of a chemical penetrating across the stratum corneum with a predicted ADME value for the design compounds in the acceptance range to be within (Table 4) the skin permeability (log Kp) for the design compounds to be within the range of −7.95 to 7.30 cm/s

The prediction of Radar plot for oral bioavailability (Table 2) indicated similar bioavailability scores for **5c–d** and **5e,i** and were found within the data range and pink color zone.

**Figure 3:**
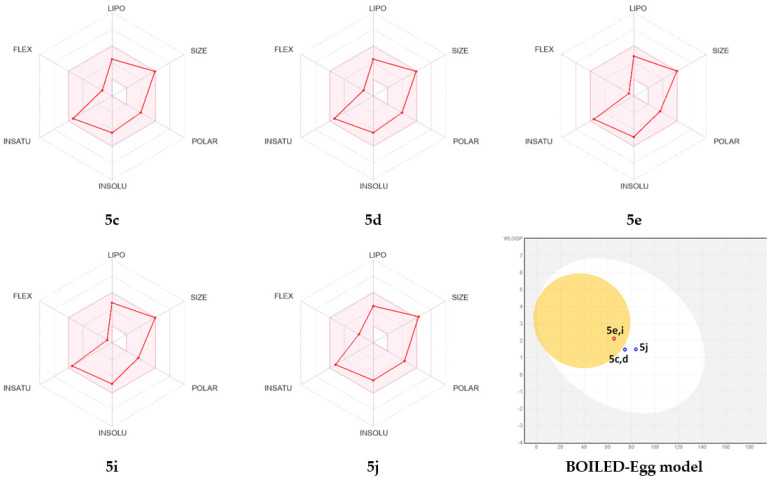
Bioavailability radar and BOILED-Egg model of potent synthesized compounds.

The inbuilt BOILED-Egg model allows for intuitive evaluation of passive gastrointestinal absorption and brain penetration in function of the position of the molecules in the WLOGP-versus-TPSA referential, revealed that **5c**, **5d** and **5j**, located in the white region for high probability of passive absorption by the gastrointestinal tract as well as no BBB penetration and P-gp positive; however, **5e** and **5i** in the yellow region are for high BBB penetration and were P-gp negative (Figure 3).

### 2.5. Molecular Docking and Dynamic Simulation

#### 2.5.1. Molecular Docking

**Figure 4:**
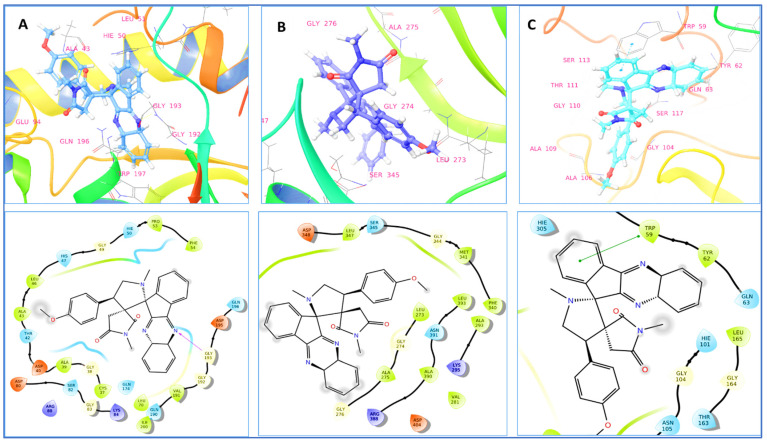
Binding interaction of promising compound **5d** with (**A**) *S. aureus* tyrosyl-tRNA synthetase (PDB ID: 1JIJ); (**B**) Tyrosine kinase (PDB ID: 2HCK). (**C**) Human pancreatic *α*-amylase (PDB ID: 2QV4).

Molecular docking analyses were carried out using the Glide software to better understand the interactions of promising compounds with *S. aureus* tyrosyl-tRNA synthetase, tyrosine kinase and human pancreatic *α*-amylase enzymes. The protein–ligand interaction is significant in structurally based drug design. In this approach, Glide docking score, emodel, glide energy score, and MMGBSA ΔG Bind are kept as support for the present work (Figure 4).

The minimum Glide energy required for the formation of complex between the ligand and the protein indicates excellent binding affinity. Very low energy indicates that the ligand is buried in the cavity of the receptor. The Glide binding energy of the promising compounds was found to be −27.625 to −46.196 kcal/mol. According to the results of the binding study, compound 5d formed one hydrogen bond with Gly193 of the *S. aureus* tyrosyl-tRNA synthetase target at a 2.70A° bond length. The methoxyphenyl and 1-methylpyrrolidine-2,5-dione portions of compound 5d were shown to have substantial van der Waals contacts with Gly38 (−1.489 kcal/mol), Asp40 (−2.166 kcal/mol), and Ala39 (−2.154 kcal/mol), which demonstrated that the molecule is entrenched within the active site (Table 5).

Complementary van der Waals interactions are shown between the co-crystallized ligand (SB-239629) and this pocket, which are delineated by the residues Ala37, Asp38, Thr40, Ala41, Ser43, Leu44, His48 and Ile101. This implies that compound **5d** could have a tyrosyl-tRNA inhibitory effect on *S. aureus* (Figure 4A). The compound **5d** is the second highest scoring compound in tyrosine kinase protein (2HCK), with a docking score of (−5.732 kcal/mol) and binding free energy of (−53.11 kcal/mol), but it was unable to connect any hydrogen bond contacts with the 2HCK receptor while making close contacts with Leu273, Gly274, Ala275 and Gly276 (Figure 4B). Compound **5d**, on the other hand, also has the best docking score (−6.182 kcal/mol) and binding free energy score (−59.16 kcal/mol) in human pancreatic *α*-amylase (2QV4). Compound **5d** in this target protein exhibits π–π interactions with hydrophobic residue Trp59 (Figure 4C). In the cavity of *α*-amylase, the amino acids Leu165 (−2.603 kcal/mol), Thr163 (−1.233 kcal/mol), Leu162 (−0.504 kcal/mol) and Gln63(−1.591 kcal/mol) stabilize the compound **5d** through non-bonded van der Waals interaction. Through van der Waals interactions with Leu162, Leu165, Tyr151, Ile148 and Ala198, the co-crystallized inhibitor also interacts significantly with a variety of hydrophobic protein residues inside the active site.

#### 2.5.2. Molecular Dynamic Simulations

**Figure 5:**
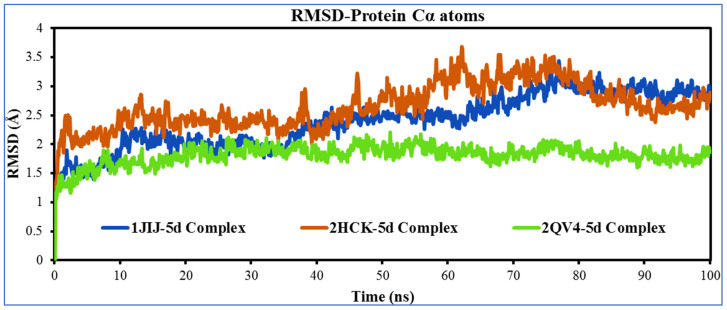
Time dependent RMSD plots of 1JIJ-**5d** complex (Blue), 2HCK-**5d** complex (red), and 2QV4-**5d** complex (green) obtained from 100 ns MD simulation Trajectories.

The molecular dynamics simulation study was conducted to understand the structural stability of the complexes. The root mean square deviations of the C*α* atoms from the docked complexes were analyzed to understand the rigidity of the complexes [52,53]. The average values of C*α* atoms RMSD for 1JIJ-**5d** complex, 2HCK-**5d** complex and 2QV4-**5d** complex were 2.400 ± 0.46 Å, 2.655 ± 0.39 Å and 1.808 ± 0.17 Å, respectively (Figure 5). The 1JIJ-**5d** complex’s RMSD pattern progressed from 2 ns to 78 ns, then displayed a steady state pattern. However, the 2HCK-5d complex had an average RMSD of 2.5 Å until 37 ns, then a rising trend with minor fluctuation until 80 ns, then the RMSD gradually decreased towards the end of the simulation. The consistency pattern of RMSD is seen in the 2QV4-5d complex, indicating more stability among the assessed complexes. All of the complexes had a maximum RMSD that was less than 3.6 Å, indicating that they were relatively stable.

**Figure 6:**
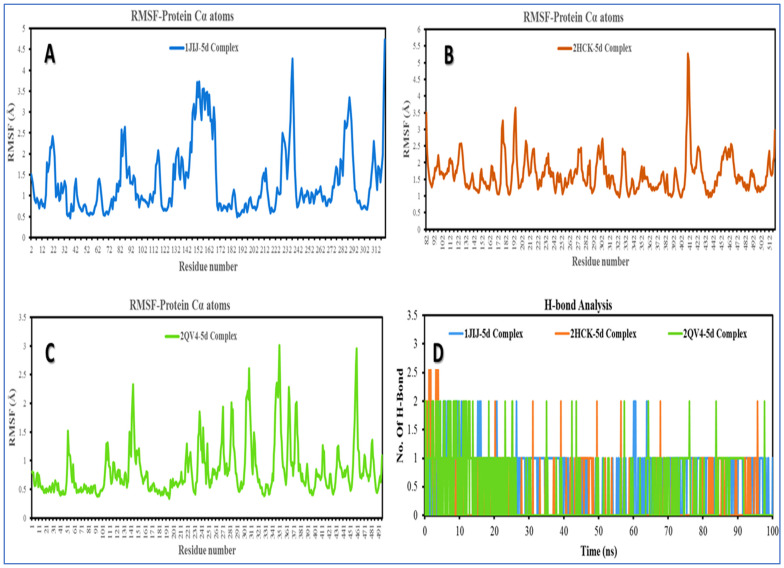
RMSF plot of (**A**). 1JIJ-5d complex (blue), (**B**). 2HCK-5d complex (red), (**C**). 2QV4-5d complex (green) and (**D**). Hydrogen bond analysis of all complexes.

To determine flexibility across the amino acid residues, the docked complexes’ root mean square fluctuations (RMSF) were examined [54,55]. With the exception of the loop region and the C-terminal, overall, the amino acids were stable during the simulation window with an RMSF range of between 0.40 Å and 4.50Å. Maximum fluctuation is seen in 1JIJ-**5d** complex with Glu236 and Ser237, with RMSF of 3.763 Å and 4.274 Å, respectively. The 1JIJ amino acids that have made contact with **5d** during the simulation trajectory include: Cys37, Gly38, Ala39, Asp40, Thr42, His47, Gly49, His50, Pro53, Phe54, Asp80, Ser82, Gly83, Lys84, Glu86, Glu87, Arg88, Val89, Tyr170, Val191, Gly193, Ser194, Asp195, Gln196, Asn199, Ile221, Leu223, Gly233, Lys234 and Trp241 (Figure 6A). All these had a lower RMSF than 1.5 Å, which indicates a lower level of flexibility of the complex. Except for the amino acids Phe 424 (3.864 Å), Lys 423 (5.036 Å) and Gly 411(3.447 Å), in the 2HCK-**5d** complex all the interacting residues exhibit a lesser magnitude of RMSF. Leu273, Gly274, Ala275 and Gly276, important residues that share hydrophobic interactions in this protein, displayed RMSFs of 1.92 Å, 2.38 Å, 2.32 Å, 2.36 Å and 2.43A Å, respectively (Figure 6B). However, only the amino acid Gly351 in the 2QV4-5d complex has an elevated RMSF of 3Å. The rest of the amino acids in this protein have a RMSF below 3 Å, which validates its stability (Figure 6C).

**Figure 7:**
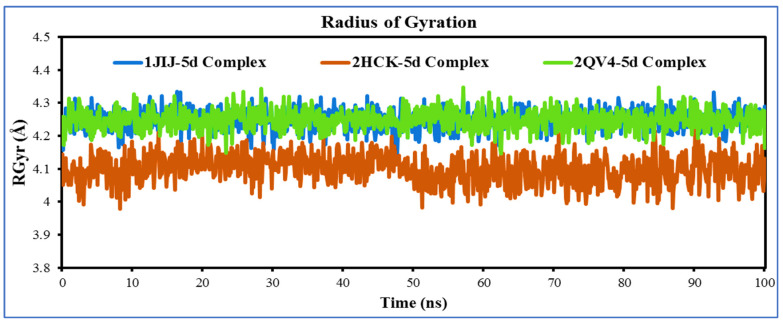
Time dependent radius of gyration (RGyr) plot of 1JIJ-5d complex (blue), 2HCK-5d complex (red) and 2QV4-5d complex (green) obtained from 100 ns MD simulation trajectories.

Hydrogen bond formation is important in stabilizing the structure of proteins and ligand–protein complexes since it minimizes the system’s energy. The ligand-protein hydrogen bonding patterns were studied in all the complexes and portrayed in graphs in comparison with time. According to hydrogen bonding data, the 2HCK-**5d** complex forms more hydrogen bonds with several critical residues at the binding site, whereas another complex forms hydrogen bonds with one to two residues. In the case of the 2QV4-**5d** complex, a maximum of two hydrogen bonds was formed, while the 1JIJ-**5d** complex, 2HCK-**5d** complex formed one hydrogen bond each (Figure 6D). However, the average number of hydrogen bonds formed with the 1JIJ-**5d** complex, 2HCK-**5d** complex and 2QV4-**5d** was 0.75, 0.20, and 0.34, respectively, which suggests that hydrogen bond formation events happen moderately with the 2HCK-**5d** complex, and it may be stabilized by hydrophobic interaction as well as ionic interaction. Assessing the radius of gyration (RGyr), which may provide information on the system’s overall tightness, is another way of determining the root mean square distance of a collection of atoms from their common center of mass [56,57]. On analyzing the RGyr plot, we have interpreted that the RGyr values of the 1JIJ-**5d** complex, 2HCK-**5d** complex and 2QV4-**5d** complex were 4.254 Å, 4.096 Å and 4.248 Å, respectively. The RGyr profiles of the complexes were stable and maintained their integrity throughout the duration of the simulation (Figure 7).

#### 2.5.3. PCA Analysis

**Figure 8:**
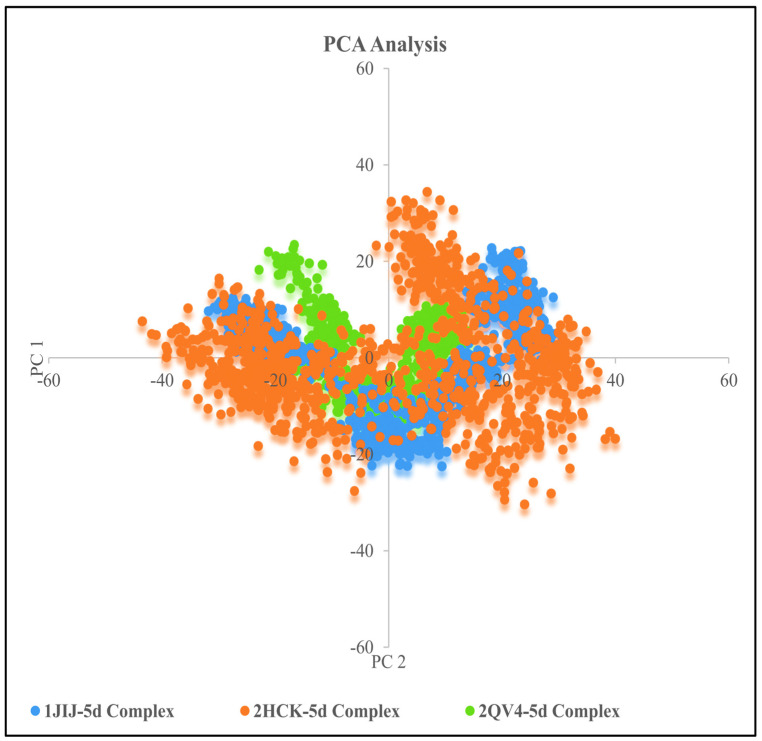
Two-dimensional projection of motion of trajectory of compound **5d** bound with proteins over the PC1 and PC2.

In order to comprehend how proteins, move in response to ligand binding, the PCA of C*α* atoms was investigated. By selecting crucial data points that indicate eigenvectors and eigenvalues that reflect the associated protein motion and the direction of motion, PCA analysis may project the high-dimensional protein dynamics into the low-dimensional space [58,59]. The PC1 and PC2 contributions for compound **5d** in a complex with the 1JIJ, 2HCK and 2QV4 systems were 42.81%, 37.14%, 16.73% and 13.06%, 12.51% and 13.24%, respectively. The data indicate that a smaller area of phase space was covered by each of the three drug–protein complexes. When compared to the 1JIJ-**5d** complex and 2HCK-**5d** complex, the 2QV4-**5d** complex in particular lowered the essential dynamics to the lowest level of functional motion. In conclusion, the correlation between the PCA results and the RMSD and RMSF results strengthens the validity of the study (Figure 8).

#### 2.5.4. MMGBSA Binding Free Energy Analysis

Any small molecule’s binding energy can reflect its affinity for a protein. MM/GBSA is a popular and reliable approach for estimating post-simulation binding free energy because it considers protein flexibility, polarizability, solvability, and entropy, which are frequently overlooked in docking procedures, and it is therefore more accurate than that of most scoring functions in use in molecular docking [59]. To explore the affinity of compound **5d** towards the 1JIJ, 2HCK and 2QV4, the binding free energy was estimated from the trajectory of MD simulation using the MMGBSA approach. The stability of the receptor-ligand complex is considered strong when the computed values of binding free energies are more negative. According to the Table 6 MMGBSA results, compound **5d** forms a stable and strong bond with the 1JIJ, 2HCK, and 2QV4; these complexes are thermodynamically favorable. In particular, binding energies of −34.15 and −45.92 kcal/mol were obtained for the 2HCK-**5d** complex and 2QV4-**5d** complex, respectively, while a much higher value was found for the 1JIJ-**5d** complex (−53.25kcal/mol); hence, the compound 5d order of affinity towards protein was as follows: 2HCK > 2QV4 > 1JIJ. Additionally, Tables S1–S3 (see supplementary materials) includes the results of computations for several additional interactions, including electrostatic energy, van der Waals interactions, nonpolar solvation energy, etc. The binding energy was revealed to be influenced adversely by solvation energy (ΔG Bind Solv GB) and favorably by Coulomb/electrostatic (ΔG Bind Coulomb) van der Waals energy (ΔG Bind VDW).

## 3. Materials and Methods

### 3.1. General Experimental Methods 

^1^H and ^13^C NMR were recorded using a Bruker spectrometer (Bruker, Bremen, Germany) operating at 400 and 100 MHz, respectively. The chemical shifts were recorded in ppm relative to tetramethylsilane and with the solvent resonance as the internal standard. Data were reported as follows: chemical shift, multiplicity (brs = broad singlet, s = singlet, d = doublet, dd = doublet of doublets, t = triplet, m = multiplet), coupling constants (Hz), integration. 13C NMR data were collected with complete proton decoupling. Chemical shifts were reported in ppm with respect to TMS with the solvent resonance as internal standard. Elemental analyses were performed on a Perkin Elmer 2400 Series II Elemental CHNS analyzer. Column chromatography was carried out on silica gel (300–400 mesh, Qingdao Marine Chemical Ltd., Qingdao, China). Thin layer chromatography (TLC) was performed on TLC silica gel 60 F254 plates 0.2 mm 200 × 200 nm. The spots were visualized using UV light at 254 nm and 360 nm.

#### General Procedure for the Preparation of Spiro-Indenoquinoxaline Pyrrolizidines **5a–m**

A mixture of ninhydrin **1** (0.5 mmol) and 1,2-phenylenediamine **2** (0.5 mmol) and sarcosine **3** (0.5 mmol) was stirred for 10 min in 10 mL of methanol followed by the addition of dipolarophile **4** (0.5 mmol). The mixture was then refluxed for 8h until completion of the reaction as evidenced by TLC. The solvent was removed under reduced pressure and the crude product obtained was purified by column chromatography on silica gel using ethylacetate-cyclohexane (3:7 *v*/*v*) as eluent to provide the pure product **5a–m**.

**5a**: Spiro [2,11″]indeno-[1,2b]-quinoxaline-spiro-[3,3′]-N-methylsuccinimide-4-(phenyl)hexahydro-1H-pyrrolidines.

White solid (105 mg, 81%); mp 173–175 °C; IR (FTIR, cm^−1^): ν = 2970, 2900, 1695, 1405. 1H NMR (400 MHz, CDCl3) δ (ppm): 2.05 (s, 3H, CH3), 2.17–2.26 (m, 2H, H4′), 2.63(s, 3H, CH3), 3.79–3.83 (t, 1H, *J* = 8 Hz, H5), 4.39–4.44 (t, 1H, *J* = 8 Hz, H5), 4.72–4.77 (t, 1H, *J* = 8 Hz, H4), 7.31–8.27 (m, 13H, Ar-H). 13C NMR (100 MHz, CDCl3) δ 24.6, 34.8, 36.3, 49.6, 59.6, 62.7, 78.9, 122.5, 127.4, 127.7, 128.9, 129.2, 129.4, 129.6, 130.1, 130.3, 131.7, 137.9, 138.0, 140.8, 142.2, 144.4, 153.7, 161.2, 174.6, 179.9. Anal. Calcd. For C29H24N4O2: C, 75.62; H, 5.21; N, 12.15. Found. C, 75.63; H, 5.20; N, 12.16.

**5b**: Spiro [2,11″]indeno-[1,2b]-quinoxaline-spiro-[3,3′]-N-methylsuccinimide-4-(4-methylphenyl)hexahydro-1H-pyrrolidines.

White solid (85 mg, 77%); mp 183–185 °C; IR (FTIR, cm^−1^): ν = 2968, 2904, 1705, 1393. 1H NMR (400 MHz, CDCl3) δ (ppm): 2.05 (s, 3H, CH3), 2.20–2.24 (m, 2H, H4′), 2.36(s, 3H, CH3), 2.63(s, 3H, CH3), 3.77–3.81 (t, 1H, *J* = 8 Hz, H5), 4.37–4.41 (t, 1H, *J* = 8 Hz, H5), 4.70–4.72 (t, 1H, *J* = 8 Hz, H4), 7.22–8.26 (m, 12H, Ar-H). 13C NMR (100 MHz, CDCl3) δ 21.1, 24.6, 34.8, 36.2, 49.3, 59.5, 62.7, 78.8, 122.5, 127.5, 129.2, 129.4, 129.6, 129.8,129.9, 130.2, 130.3, 131.6, 138.0, 134.2, 134.7, 137.4, 137.9, 140.8, 153.7, 161.2, 174.7, 179.9. Anal. Calcd. For C30H26N4O2: C, 75.92; H, 5.47; N, 11.80. Found. C, 75.94; H, 5.50; N, 11.81.

**5c**: Spiro [2,11″]indeno-[1,2b]-quinoxaline-spiro-[3,3′]-N-methylsuccinimide-4-(2 methoxyphenyl)hexahydro-1H-pyrrolidines.

White solid (102 mg, 80%); mp 196–198 °C; 1H NMR (400 MHz, CDCl3) δ (ppm): 2.04 (s, 3H, CH3), 2.37–2.45(d, 1H, *J* = 9 Hz, H4′), 2.86 (s, 3H, CH3), 3.63 (s, 3H, OCH3), 3.73–3.77 (t, 1H, *J* = 8 Hz, H5), 4.63–4.67 (t, 1H, *J* = 8 Hz, H5), 4.79–4.84 (t, 1H, *J* = 12 Hz, H4), 6.82–8.19 (m, 12H, Ar-H). 13C NMR (100 MHz, CDCl3) δ 24.7, 35.1, 35.7, 43.1, 55.1, 56.6, 60.6, 78.9, 120.7, 122.3, 126.0, 127.5, 128.5, 129.1, 129.2, 129.5, 130.0, 130.1, 130.4, 132.0, 138.6, 141.0, 141.9, 143.7, 153.3, 162.0, 175.5, 181.1. Anal. Calcd. For C30H26N4O3: C, 73.44; H, 5.30; N, 11.41. Found. C, 73.45; H, 5.31; N, 11.42.

**5d**: Spiro [2,11″]indeno-[1,2b]-quinoxaline-spiro-[3,3′]-N-methylsuccinimide-4-(4-methoxyphenyl)hexahydro-1H-pyrrolidines.

White solid (69 mg, 65%); mp 179–181 °C; IR (FTIR, cm^−1^): ν = 2984, 2903, 1692, 1393. 1H NMR (400 MHz, CDCl3) δ (ppm): 2.10 (s, 3H, CH3), 2.35–2.46 (m, 2H, H4′), 2.59(s, 3H, CH3), 3.72–3.84 (t, 2H, *J* = 8 Hz, H5), 3.91 (s, 3H, OCH3), 4.89–4.94(t, 1H, *J* = 8 Hz, H4), 7.34–8.10 (m, 12H, Ar-H). 13C NMR (100 MHz, CDCl3) δ 24.1, 31.7, 34.5, 48.0, 52.5, 55.4, 61.6, 79.3, 122.5, 124.8, 127.2, 128.9, 129.1, 129.5, 130.1, 130.3, 130.7, 131.6, 132.4, 132.5, 132.9, 133.5, 136.8, 140.3, 152.6, 161.0, 174.6, 177.4. Anal. Calcd. For C30H26N4O3: C, 73.44; H, 5.30; N, 11.41. Found. C, 73.45; H, 5.31; N, 11.40.

**5e**: Spiro [2,11″]indeno-[1,2b]-quinoxaline-spiro-[3,3′]-N-methylsuccinimide-4-(4-chlorophenyl)hexahydro-1H-pyrrolidines.

White solid (80 mg, 75%); mp 182–184 °C; 1H NMR (400 MHz, CDCl3) δ (ppm): 2.11 (s, 3H, CH3), 2.36–2.41 (m, 2H, H4′), 2.59 (s, 3H, CH3), 3.67–3.72(m, 2H, H5), 4.69–4.75 (t, 1H, *J* = 12 Hz, H4), 7.31–8.14 (m, 12H, Ar-H). 13C NMR (100 MHz, CDCl3) δ 24.2, 31.7, 34.5, 48.0, 52.4, 61.6, 79.1, 122.4, 127.0, 128.0, 128.3, 129.0, 129.1, 129.6, 130.1, 131.3, 130.7, 131.6, 132.1, 132.4, 138.8, 142.9, 152.5, 160.9, 174.3, 178.7. Anal. Calcd. For C29H23ClN4O2: C, 70.36; H, 4.64; N, 11.31 Found. C, 70.38; H, 4.65; N, 11.29.

**5f**: Spiro [2,11″]indeno-[1,2b]-quinoxaline-spiro-[3,3′]-N-methylsuccinimide-4-(4-bromophenyl)hexahydro-1H-pyrrolidines.

White solid (52 mg, 68%); mp 200–202 °C; 1H NMR (400 MHz, CDCl3) δ (ppm): 2.04 (s, 3H, CH3), 2.08–2.11 (m, 2H, H4′), 2.65 (s, 3H, CH3), 3.80–3.84(t, 1H, *J* = 8Hz, H5), 4.33–4.38 (t, 1H, *J* = 12 Hz, H4), 4.65–4.70(t, 1H, *J* = 12 Hz, H4), 7.50–8.24 (m, 12H, Ar-H). 13C NMR (100 MHz, CDCl3) δ 24.7, 34.7, 36.3, 49.0, 59.9, 62.5, 78.9, 122.6, 127.2, 129.3, 129.4, 130.4, 1130.5, 131.3, 131.8, 131.9, 132.1, 132.6, 137.1, 140.7, 142.2, 144.1, 153.7, 161.2, 174.2, 179.9. Anal. Calcd. For C29H23BrN4O2: C, 64.56; H, 4.26; N, 10.38. Found. C, 64.57; H, 4.28; N, 10.39.

**5g**: Spiro [2,11″]indeno-[1,2b]-quinoxaline-spiro-[3,3′]-N-methylsuccinimide-4-(4-fluorophenyl)hexahydro-1H-pyrrolidines.

White solid (83 mg, 71%); mp 205–207 °C; ^1^H NMR (400 MHz, CDCl3) δ (ppm): 2.04 (s, 3H, CH3), 2.10–2.38 (m, 2H, H4′), 2.64 (s, 3H, CH3), 3.80–3.84(t, 1H, *J* = 8 Hz, H5), 4.33–4.38 (t, 1H, *J* = 12 Hz, H5), 4.68–4.73 (t, 1H, *J* = 8 Hz, H4), 7.09–8.23 (m, 12H, Ar-H). 13C NMR (100 MHz, CDCl3) δ 24.6, 34.7, 36.3, 48.9, 60.1, 62.6, 78.9, 122.5, 127.3, 129.3, 129.5, 130.4, 131.7, 131.8, 132.0, 132.1, 133.0, 138.0, 140.7, 144.2, 153.7, 161.3, 174.3, 180.0. Anal. Calcd. For C29H23FN4O2: C, 72.78; H, 4.80; N, 11.70. Found. C, 72.77; H, 4.81; N, 11.71.

**5h**: Spiro [2,11″]indeno-[1,2b]-quinoxaline-spiro-[3,3′]-N-methylsuccinimide-4-(4-thiophenyl)hexahydro-1H-pyrrolidines.

White solid (52 mg, 59%); mp 203–205 °C; ^1^H NMR (400 MHz, CDCl3) δ (ppm): 2.10 (s, 3H, CH3), 2.33–2.36 (d, 1H, *J* = 12 Hz, H4′), 2.62 (s, 3H, CH3), 3.09–3.14 (d, 1H, *J* = 20, H4′), 3.68–3.74 (m, 2H, H5), 4.69 (t, 1H, *J* = 8 Hz, H4), 7.20–8.27 (m, 12H, Ar-H). 13C NMR (100 MHz, CDCl3) δ 24.2, 31.9, 34.5, 48.8, 52.4, 61.4, 79.2, 122.5, 124.7, 127.1, 128.9, 129.5, 129.7, 130.2, 130.9, 131.6, 132.5, 132.8, 136.8, 138.5, 142.2, 143.1, 152.6, 161.0, 174.5, 178.6. Anal. Calcd. For C27H22N4O2 S: C, 69.50; H, 4.71; N, 12.00. Found. C, 69.49; H, 4.72; N, 12.02.

**5i**: Spiro [2,11″]indeno-[1,2b]-quinoxaline-spiro-[3,3′]-N-methylsuccinimide-4-(3-chloro-phenyl)hexahydro-1H-pyrrolidines.

White solid (96 mg, 70%); mp 193–195 °C; 1H NMR (300 MHz, CDCl3) δ (ppm): 2.04 (s, 3H, CH3), 2.10–2.12(m, 2H, H4′), 3.82–3.88 (t, 1H, *J* = 9 Hz, H5), 4.34–4.40 (t, 1H, *J* = 9.3 Hz, H5), 4.63–4.69 (t, 1H, *J* = 8.7 Hz, H4), 7.32–8.38 (m, 12H, Ar-H). 13C NMR (75 MHz, CDCl3) δ 24.6, 31.5, 34.8, 49.1, 52.9, 62.4, 79.7, 122.5, 124.8, 126.3, 126.6, 129.0, 129.6, 130.3, 130.5, 130.8, 131.6, 132.4, 132.5, 134.3, 134.9, 136.8, 141.5, 156.6, 161.2, 174.4, 177.0. Anal. Calcd. For C29H23ClN4O2: C, 70.36; H, 4.64; N, 11.31. Found. C, 70.33; H, 4.68; N, 11.30.

**5j**: Spiro [2,11″]indeno-[1,2b]-quinoxaline-spiro-[3,3′]-N-methylsuccinimide-4-(3,4- dimethoxyphenyl)hexahydro-1H-pyrrolidines.

White solid (79 mg, 63%); mp 211–213 °C; 1H NMR (400 MHz, CDCl3) δ (ppm): 2.10 (s, 3H, CH3), 2.36–2.38(d, 1H, *J* = 8 Hz, H4′), 2.41 (s, 3H, CH3), 3.66–3.69 (d, 1H, *J* = 12 Hz, H4′), 3.70–3.73 (m, 2H, H5), 4.01 (s, 6H, 2(OCH3)), 4.50–4.54 (t, 1H, *J* = 8 Hz, H4), 6.95–8.22 (m, 11H, Ar-H). 13C NMR (100 MHz, CDCl3) δ 24.2, 31.5, 34.5, 49.9, 52.5, 55.9, 56.0, 61.8, 79.4, 122.3, 126.5, 126.9, 127.2, 128.9, 129.0, 129.1, 129.5, 130.1, 130.2, 130.6, 138.6, 140.5, 141.3, 142.1, 143.2, 148.4, 150.8, 161.1, 174.6, 181.4. Anal. Calcd. For C31H28N4O4: C, 71.51; H, 5.37; N, 10.75. Found. C, 71.51; H, 5.36; N, 11.76.

**5k**: Spiro [2,11″]indeno-[1,2b]-quinoxaline-spiro-[3,3′]-N-methylsuccinimide-4-(3-bromo-4-methoxyphenyl)hexahydro-1H-pyrrolidines.

White solid (68mg, 73%); mp 209–211 °C; 1H NMR (400 MHz, CDCl3) δ (ppm): 2.10 (s, 3H, CH3), 2.33–2.38 (m, 2H, H4′), 2.59 (s, 3H, CH3), 3.64–3.72 (m, 2H, H5), 4.00 (s, 3H, OCH3), 4.87–4.93 (t, 1H, *J* = 8 Hz, H4), 6.99–8.22 (m, 11H, Ar-H). 13C NMR (100 MHz, CDCl3) δ 24.2, 31.7, 34.5, 49.4, 52.4, 56.3, 61.6, 79.2, 122.3, 127.0, 127.8, 128.9, 129.1, 129.6, 130.1, 130.7, 132.2, 136.6, 138.5, 141.2, 142.2, 143.0, 152.6, 161.0, 174.4, 177.8. Anal. Calcd. For C30H25BrN4O3: C, 63.27; H, 4.39; N, 9.83. Found. C, 63.28; H, 4.41; N, 9.84.

**5l**: Spiro [2,11″]indeno-[1,2b]-quinoxaline-spiro-[3,3′]-N-methylsuccinimide-4-(3-bromo-4-hydroxy-phenyl)hexahydro-1H-pyrrolidines.

White solid (73 mg, 64%); mp 189–191 °C; 1H NMR (400 MHz, CDCl3) δ (ppm): 2.09 (s, 3H, CH3), 2.16–2.28(m, 2H, H4′), 2.62 (s, 3H, CH3), 3.75–3.81 (t, 1H, *J* = 8 Hz, H5), 4.38–4.43 (t, 1H, *J* = 8 Hz, H5), 4.71–4.75 (t, 1H, *J* = 8 Hz, H4), 7.42–8.29 (m, 11H, Ar-H). 13C NMR (100 MHz, CDCl3) δ 24.6, 34.8, 36.4, 49.1, 59.7, 62.8, 79.0, 122.5, 125.8, 126.1, 127.4, 129.2, 129.4, 129.6, 129.8, 130.3, 131.7, 134.8, 137.9, 140.8, 142.2, 144.5, 150.6, 153.7, 161.3, 174.8, 180.0. Anal. Calcd. For C29H23BrN4O3: C, 62.70; H, 4.14; N, 10.08. Found. C, 62.74; H, 4.15; N, 10.11.

**5m**: Spiro [2,11″]indeno-[1,2b]-quinoxaline-spiro-[3,3′]-N-methylsuccinimide-4-(3,5-dibromo-4-hydroxyphenyl)hexahydro-1H-pyrrolidines.

White solid (98 mg, 80%); mp 214–216 °C; 1H NMR (400 MHz, CDCl3) δ (ppm): 2.00 (s, 3H, CH3), 2.47–2.51 (m, 2H, H4′), 2.69 (s, 3H, CH3), 3.82–3.87 (t, 1H, *J* = 8 Hz, H5), 4.23–4.28 (t, 1H, *J* = 12 Hz, H5), 4.49–4.55 (t, 1H, *J* = 8 Hz, H4), 7.57–8.45 (m, 10H, Ar-H). 13C NMR (100 MHz, CDCl3) δ 24.7, 34.5, 36.6, 48.3, 61.1, 62.6, 78.8, 122.6, 125.7, 126.8, 129.3, 129.6, 130.5, 130.6, 131.9, 133.2, 134.0, 138.2, 140.7, 142.2, 143.8, 148.9, 153.5, 161.4, 174.1, 180.3. Anal. Calcd. For C29H22 Br2N4O3: C, 54.86; H, 3.46; N, 8.82. Found. C, 54.89; H, 3.47; N, 8.84.

### 3.2. Pharmacological Study

#### 3.2.1. Antimicrobial Activity

In the present study, the antimicrobial activity of the synthesized compounds was screened by agar disc diffusion method according to the protocol described by Kadri et al. [49], against four bacteria, namely *Staphylococcus aureus* ATCC 25923, *Micrococccus luteus* NCIMB 8166, *Escherichia coli* ATCC 25,922 and *Pseudomonas aeruginosa* ATCC 27853. Moreover, it was tested against two fungal strains, namely *Candida albicans* ATCC 90,028 and *Candida krusei* ATCC 6258. The inoculums of the microorganisms were adjusted to 0.1 at OD600 for bacteria and 0.4 at OD540 for yeasts) and then streaked onto Muller–Hinton (MH) and Sabouraud (SB) agar plates using a sterile cotton mop. Sterile filter discs (diameter 6 mm, Biolife Italy) were placed at the surface of the appropriate agar media and 10 µL of the product dissolved in 10% of solvent was dropped onto each disc. Tetracycline (10 mg/mL; 10 μL/disc) and amphotericin B (10 mg/mL; 10 μL/disc) were used as reference antibiotics. After incubation at 37 °C for 24h, the antibacterial activities were evaluated by measuring an inhibition zone formed around the disc. Each assay was performed in triplicate.

#### 3.2.2. Antioxidant Activity

DPPH free Radical Scavenging Activity.

DPPH, the stable artificial free radicals, has been widely used for the measurement of free radical scavenging capacity of the compounds in ethanol and aqueous systems [60]. Briefly, 2 mL DPPH solution (0.2 mM, in 95% ethanol) was incubated with 2 mL different concentrations of compounds solution. Then, the reaction mixture was shaken and incubated in the dark for 40 min at room temperature. The absorbance was immediately recorded at 517 nm against ethanol with a spectrophotometer (Metash, model UV-5200, Shanghai Xiwen Biotech. Co., Ltd, Shanghai, China). The DPPH free radical scavenging rate was calculated using the equation:

DPPH scavenging activity (%) = A_0_ − A_1_/A_0_ × 100

where A_0_ was the absorbance of the control reaction (containing all reagents except the tested compound), and A1 was the absorbance of the test reaction (containing all reagents with the tested compound). The percentage of DPPH radical scavenging activity was plotted against the sample concentration to acquire the IC_50_ value, defined as the concentration of sample necessary to cause 50% inhibition. Radical scavenging activity (RSA) was calculated from the IC_50_ value as the equation: RSA  =  pIC_50_  =  −lg[IC_50_]. The smaller the IC_50_ value, the larger is the RSA value and the higher is the antioxidant activity.

Radical Scavenging Activity ABTS.

The ABTS assay was performed as previously described by Tuberoso et al. [61]. ABTS^+^ radical was generated by oxidation of ABTS with potassium persulfate. The blue–green ABTS was produced through the reaction between 2 mM ABTS and 70 mM potassium persulfate in water. The mixture was left to stand in the dark for 12 to 16 h before use. The ABTS solution was diluted with methanol to an absorbance of 0.700 ± 0.005 at 734 nm. Then 2 mL of diluted ABTS solution was mixed with 100 μL of samples and absorbance was measured after 1 min incubation at room temperature. A standard curve was obtained by using Trolox as standard solution. The results were expressed as μM Trolox equivalent. 

The mixture of 7 mmol/L aqueous ABTS solution with 2.45 mmol/L potassium persulfate solution constitutes a stable ABTS^+^ stock solution that was allowed to stand in the dark and at room temperature for 12 to 16 h before use. A 1 mL sample of the diluted ABTS solution (adjusted to 0.7 ± 0.02 at 743 nm) was added to 10 μL of each product. The absorbance was determined after 6 min. The percentage inhibition of the ABTS˙ radical was calculated from the following equation:

% inhibition = (Abs_control_ − Abs_test_)/Abs_control_ × 100

Abs control: the control absorbance.

Abs test: the absorbance of the test sample after 6 min.

Ferric reducing power assay (FRAP).

The FRAP assay was used to determine the AC of the products by the reduction of Fe(III) and Fe (II). Briefly, Fe(III) was reduced to Fe(II), and Fe(II) was mixed with TPTZ to form a blue complex with strong absorption peak at 593 nm at pH  =  3.6 condition. Acetate buffer (pH = 3.6), TPTZ solution (10 mM, in 40 mM hydrochloric acid) and FeCl_3_ solution (20 mM, in water) were mixed in a ratio of 10:1:1 to prepare FRAP working solution. The products solution (0.5 mL) was mixed with 4.0 mL FRAP working solution, and reacted at 37 °C for 30 min in the dark, and the absorbance at 593 nm was immediately recorded with a spectrophotometer. The result was expressed as the equivalent amount of Trolox per mole of the samples (mol TE/mol) [62].

#### 3.2.3. α-Amylase Inhibitory Assay

The inhibition of *α*-amylase enzyme assays was performed according to the protocol described by Amamou et al. [63]. Each compound was dissolved in the minimum of DMSO at different concentrations and then diluted in sodium phosphate buffer (0.02 M, pH 6.9, with 0.006 M NaCl). The DMSO level did not exceed 1% in the mixture. After that, 50 μL of each sample solution was added to 50 μL of *α*-amylase solution (0.5 mg/mL dissolved in the same buffer medium. After pre-incubation for 10 min at 25 °C, 50 μL of the starch solution (1% in buffer solution) was added to the mixture. After incubation at 25 °C for 10 min, the reaction was mixed with 100 μL of 3,5-dinitrosalicylic acid (DNS) solution. At this point, the test tubes were placed in a boiling water bath for 5 min, followed by cooling to room temperature. Next, each solution was diluted with 1 mL of distilled water. Over time, the absorbance was measured at 540 nm by Thermo ScientificTM MultiskanTM GO Microplate Spectrophotometer (Thermo Fisher Scientific Oy, Ratastie, Finland) using a 96-well microplate.

The *α*-amylase inhibitory activity was determined as follows:

Inhibition (%) = 100 [(Abs_Control_ − Abs_Sample_)/(Abs_Control_)]

### 3.3. Computational Study

#### 3.3.1. Molecular Docking

Molecular docking was conducted in order to assess the binding interactions of the promising compounds. Initially, by using the LigPrep module synthesized compounds, their structures were transformed from 2D to low-energy 3D conformers with satisfactory bond lengths and angles. The 3D structures of *S. aureus* tyrosyl-tRNA synthetase, tyrosine kinases, and human pancreatic *α*-amylase were obtained from the PDB database under the accession codes 1JIJ, 2HCK and 2QV4, respectively. Before docking, all protein crystal structures were prepared using the Protein Preparation wizard tool by Schrodinger to address any structural problems. This process involves changing the bond order, adding hydrogens, looking for disulfide bonds and filling in side chains and loops that are lacking [64,65]. Constrained minimization was additionally applied to the protein structure. In which, heavier atoms in the structure are constrained to reduce torsional stress throughout this reduction phase, while hydrogens are left unconstrained. Using the Schrodinger Receptor Grid Generation tool, a crystalized ligand structure was selected to generate a 3D grid with accurate dimensions to represent the active part of the receptor [66]. The binding free energy of protein–ligand complexes was calculated using Prime of Schrodinger.

#### 3.3.2. Molecular Dynamics (MD) Simulation

Protein–ligand complexes that showed good binding interactions were subjected to MD studies using Desmond for 100 ns [66]. The chloride and sodium ions are added to neutralize the net charge of the system to zero, and the system is submerged in an orthorhombic box (10 Å × 10 Å ×10 Å) filled with SPC water molecules [67,68]. The MD job was performed using an NPT ensemble at a 300 K temperature and 1.01325 bar pressure, which were kept throughout the simulation. To assess the stability of the docked ligands for each system, 1000 trajectories were collected during the simulations and evaluated with different parameters [69]. The conformational ensemble (1000 snapshots collected from 0 to 100 ns) acquired from the MD simulations was subjected to principal component analysis (PCA). By creating a covariance matrix and addressing the atomic movements responsible for the conformational changes, PCA aids in capturing the displacement of Cα atoms. PCA with the trj essential dynamic command was used to investigate protein-ligand confirmations and major global movements following ligand binding. The complete MD simulations were accomplished in the Z2 TWR G4 WKS, Linux (Ubuntu 18.04.3 LTS 64-bit) environment with a CPU Core i7-9700, DDR4-2666 nECC RAM and a NVIDIA Quadro P620/PCIe/SSE2 CUDA core GPU.

#### 3.3.3. ADME Study

Pharmacokinetic properties of the titled compounds were predicted using ADME (absorption, distribution, metabolism and excretion) descriptors by a SwissADME online server (http://www.swissadme.ch/ assessed on 29 July 2022) [70,71].

## 4. Conclusions

The present report details the design and synthesis of a novel series of spiropyrrolidine tethered indeno-quinoxaline heterocyclic hybrids as potent antimicrobial, antioxidant and antidiabetic agents. The structures of the aforementioned compounds were confirmed using different spectroscopic techniques. These designed compounds were evaluated in vitro for their antimicrobial, antioxidant and antidiabetic potential. The results demonstrated that most of the tested compounds showed potent antibacterial and antifungal activities towards pathogenic strains. Interestingly, these analogues also exhibited remarkable α-amylase inhibitory effects. Molecular docking and molecular dynamics simulation studies revealed that compound 5d in complex with *S. aureus* tyrosyl-tRNA synthetase, Tyrosine kinase, and human pancreatic α-amylase have good docking and molecular dynamics profiles. Some of the studied compounds can be considered promising lead compounds in the design of more potent dual antimicrobials and antidiabetic agents.

## Figures and Tables

**Table 1:** Optimization of the reaction conditions ^a^.

Entry	Solvent	T (°C)	Time (h)	Yield ^b^ (%)
1	CH_3_CN	25	8	-
2	CH_3_CN	80	4	35
3	CH_3_CN	80	12	43
4	MeOH	25	8	-
5	MeOH	64	4	81
6	MeOH	64	24	81

^a^ The reactions were carried out with **1** (0.5 mmol), **2** (0.5 mmol), **3** (0.75mmol) and **4a** (0.5 mmol) in solvent (5 mL). ^b^ Overall yields after isolation of the products by column chromatography. ^-^ No reaction due to insufficient solubility.

**Table 2:** MIC, MBC, MFC, MBC/MIC and MFC/MIC of the synthesized compounds against the tested pathogenic strains.

Entry	MBC (mM), MFC (mM), MBC/MIC and MFC/MIC																	
	Gram-Positive Bacteria						Gram-Negative Bacteria						Fungi					
	*S. aureus* ATCC 25923			*M. luteus* NCIMB 8166			*E. coli* ATCC 25922			*P. aeruginosa* ATCC 27853			*C. albicans* ATCC 90028			*C. krusei* ATCC 6258		
	MIC	MBC	MBC/ MIC	MIC	MBC	MBC/MIC	MIC	MBC	MBC/MIC	MIC	MBC	MBC/MIC	MIC	MFC	MFC/MIC	MIC	MFC	MFC/MIC
**5a**	135.12	270.24	2	135.12	-	-	270.24	540.49	2	135.12	540.49	4	135.12	135.12	1	67.56	67.56	1
**5b**	65.57	524.58	8	524.58	-	-	262.29	524.58	2	131.14	524.58	4	131.14	262.29	2	65.57	65.57	1
**5c**	31.71	507.54	16	126.88	-	-	253.77	507.54	4	253.77	507.54	2	63.44	63.44	1	63.44	63.44	1
**5d**	3.95	126.88	32	63.44	253.77	4	126.88	507.54	4	126.88	507.54	4	31.71	126.88	4	15.85	15.85	1
**5e**	15.71	251.51	16	251.51	251.51	1	251.51	503.02	2	125.75	503.02	4	62.87	62.87	1	31.42	31.42	1
**5f**	115.43	230.86	2	230.86	-	-	230.86	461.73	2	115.43	230.86	2	57.71	57.71	1	57.71	57.71	1
**5g**	16.25	260.12	16	130.06	-	-	260.12	520.25	2	130.06	260.12	2	65.03	65.03	1	32.50	32.50	1
**5h**	33.33	533.53	16	133.38	133.38	1	266.76	533.53	2	133.38	266.76	2	66.69	266.76	4	66.69	66.69	1
**5i**	125.75	503.02	4	251.51	251.51	1	251.51	503.02	2	125.75	503.02	4	125.75	503.02	-	31.42	31.42	1
**5j**	14.94	478.38	32	239.19	478.38	2	239.19	478.38	2	239.19	478.38	2	119.59	239.19	2	59.79	59.79	1
**5k**	54.68	437.47	8	218.73	-	-	218.73	437.47	2	218.73	437.47	2	218.73	218.73	1	54.68	54.68	1
**5l**	28.02	448.47	16	112.11	224.23	2	224.23	448.47	2	112.11	448.47	4	56.05	448.47	8	28.02	28.02	1
**5m**	24.54	196.43	8	98.21	-	-	196.43	196.43	1	98.21	392.87	4	98.21	98.21	1	49.10	49.10	1
**Tetracycline**	576.01	288.00	2	576.01	1152.02	2	281.25	288.00	1	576.01	1152.02	2	-	-	-	-	-	-
**Amphotericin B**		-			-	-		-	-		-	-	5.41	5.41	1	5.41	5.41	1

-: No activity.

**Table 3:** In vitro antioxidant and antidiabetic activities of the synthesized compounds.

Entry	IC_50_ (mM)			
	DPPH	ABTS	FRAP	*α*-Amylase
**5a**	15.36 ± 0.65	49.19 ± 0.46	17.02 ± 0.52	1.19 ± 0.02
**5b**	33.74 ± 0.004	57.51 ± 0.55	63.26 ± 0.21	2.00 ± 0.30
**5c**	3.26 ± 0.32	7.03 ± 0.07	3.69 ± 0.72	0.92 ± 0.10
**5d**	7.44 ± 0.15	9.78 ± 0.30	8.09 ± 0.82	0.55 ± 0.38
**5e**	6.12 ± 0.01	12.16 ± 0.18	6.54 ± 0.18	1.91 ± 0.37
**5f**	16.13 ± 0.39	18.12 ± 0.53	7.93 ± 0.44	1.69 ± 0.28
**5g**	26.03 ± 0.50	7.19 ± 0.11	20.54 ± 0.60	1.94 ± 0.37
**5h**	19.13 ± 0.23	18.04 ± 0.13	6.32 ± 0.68	1.90 ± 0.054
**5i**	7.80 ± 0.32	11.39 ± 0.36	5.98 ± 0.52	1.33 ± 0.72
**5j**	19.20 ± 0.19	11.56 ± 0.05	5.98 ± 0.17	0.95 ± 0.14
**5k**	123.44 ± 0.45	7.69 ± 0.36	6.03 ± 0.12	2.19 ± 0.23
**5l**	15.28 ± 0.37	15.19 ± 0.31	3.26 ± 0.45	2.16 ± 0.35
**5m**	47.29 ± 0.06	14.56 ± 0.26	7.38 ± 0.14	1.40 ± 0.11
**Trolox**	31.24 ± 3.67	99.88 ± 0.31	41.87 ± 2.07	-
**Acarbose**	-	-	-	1.19 ± 0.02

**Table 4:** ADME properties of potent synthesized compounds.

Entry	5c	5d	5e	5i	5j
Physicochemical Properties/Lipophilicity/Druglikeness					
Molecular weight	492.57	492.57	496.99	496.99	522.59
Num. heavy atoms	37	37	36	36	39
Num. arom. heavy atoms	12	12	12	12	12
Num. rotatable bonds	0.33	0.33	0.31	0.31	0.35
Num. H-bond acceptors	2	2	1	1	3
Num. H-bond donors	6	6	5	5	7
Molar Refractivity	0	0	0	0	0
TPSA	155.61	155.61	154.12	154.12	162.10
Consensus Log Po/w	2.77	2.76	3.21	3.29	2.73
Lipinski’s Rule	Yes	Yes	Yes	Yes	Yes
Bioavailability Score	0.55	0.55	0.55	0.55	0.55
Pharmacokinetics					
GI absorption	High	High	High	High	High
BBB permeant	No	No	Yes	Yes	No
P-gp substrate	Yes	Yes	No	No	Yes
CYP1A2 inhibitor	No	No	Yes	Yes	No
CYP2C19 inhibitor	Yes	Yes	Yes	Yes	Yes
CYP2C9 inhibitor	No	No	No	No	No
CYP2D6 inhibitor	No	No	No	No	No
CYP3A4 inhibitor	Yes	Yes	Yes	Yes	Yes
Log Kp (cm/s)	−7.74	−7.74	−7.30	−7.30	−7.95

**Table 5:** Glide dock score and binding free energy of promising compounds with targeted proteins.

Compound	Docking Score	Glide Emodel	Glide Energy	Prime Energy	MMGBSA ΔG Bind
	***S. aureus* Tyrosyl-tRNA Synthetase (1JIJ)**				
**5d**	−6.843	−57.042	−46.196	−12980.2	−43.02
**5e**	−4.834	−30.317	−27.625	−12967.4	−20.76
**5j**	−5.529	−56.637	−44.992	−12980.4	−47.93
Reference ^a^	−7.973	−98.597	−68.426	13103.87	−63.92
	**Tyrosine Kinase (2HCK)**				
**5e**	−5.804	−41.828	−39.753	−17858.1	−58.03
**5d**	−5.732	−45.891	−37.914	−17853	−53.11
**5i**	−5.288	−41.24	−36.51	−17847.7	−54.74
**5c**	−5.022	−32.357	−30.204	−17827.1	−34.18
Reference ^a^	−8.551	−67.224	−45.767	−17945.56	−59.30
	**Human Pancreatic *α*-Amylase (2QV4)**				
**5d**	−6.182	−41.759	−35.02	−22748.8	−59.16
**5c**	−6.172	−36.006	−33.572	−22749.8	−51.25
**5j**	−6.146	−46.534	−38.443	−22751.3	−56.64
Reference ^a^	−8.141	−99.527	−74.699	−22821.47	−76.71

**^a^** co-crystalized ligand.

**Table 6:** The Prime MMGBSA (ensemble-averaged) binding free energies (kcal/mol) of compound **5d** with targeted proteins during the 100 ns MD simulation.

MMGBSA Components (kcal/mol)	1JIJ-5d Complex	2HCK-5d Complex	2QV4-5d Complex
ΔG Bind	−53.25	−34.15	−45.92
ΔG Bind Coulomb	−9.72	−6.28	−3.55
ΔG Bind H bond	−0.54	−0.19	−0.33
ΔG Bind Lipo	−18.56	−12.93	−20.37
ΔG Bind Solv GB	34.23	19.24	16.57
ΔG Bind VDW	−58.70	−34.61	−37.90

## Data Availability

Not applicable.

## Supplementary Materials

The following supporting information can be downloaded at: https://www.mdpi.com/article/10.3390/molecules27217248/s1. Figure S1. ^1^H NMR (CDCl_3_) spectrum of Compound (**5a**), Figure S2. ^13^C NMR (CDCl_3_) spectrum of Compound (**5a**), Figure S3. ^1^H NMR (CDCl_3_) spectrum of Compound (**5b**), Figure S4. ^13^C NMR (CDCl_3_) spectrum of Compound (**5b**), Figure S5. ^1^H NMR (CDCl_3_) spectrum of Compound (**5c**), Figure S6. ^13^C NMR (CDCl_3_) spectrum of Compound (**5c**) Figure S7. ^1^H NMR (CDCl_3_) spectrum of Compound (**5d**), Figure S8. ^13^C NMR (CDCl_3_) spectrum of Compound (**5d**), Figure S9. ^1^H NMR (CDCl_3_) spectrum of Compound (**5e**), Figure S10. ^13^C NMR (CDCl_3_) spectrum of Compound (**5e**), Figure S11. ^1^H NMR (CDCl_3_) spectrum of Compound (**5f**), Figure S12. ^13^C NMR (CDCl_3_) spectrum of Compound (**5f**), Figure S13. ^1^H NMR (CDCl_3_) spectrum of Compound (**5g**), Figure S14. ^13^C NMR (CDCl_3_) spectrum of Compound (**5g**), Figure S15. ^1^H NMR (CDCl_3_) spectrum of Compound (**5h**), Figure S16. ^13^C NMR (CDCl_3_) spectrum of Compound (**5h**), Figure S17. ^1^H NMR (CDCl_3_) spectrum of Compound (**5i**), Figure S18. ^13^C NMR (CDCl_3_) spectrum of Compound (**5i**), Figure S19. ^1^H NMR (CDCl_3_) spectrum of Compound (**5j**), Figure S20. ^13^C NMR (CDCl_3_) spectrum of Compound (**5j**), Figure S21. ^1^H NMR (CDCl_3_) spectrum of Compound (**5k**), Figure S22. ^13^C NMR (CDCl_3_) spectrum of Compound (**5k**), Figure S23. ^1^H NMR (CDCl_3_) spectrum of Compound (**5l**), Figure S24. ^13^C NMR (CDCl_3_) spectrum of Compound (**5l**), Figure S25. ^1^H NMR (CDCl_3_) spectrum of compound (**5m**), Figure S26. ^13^C NMR (CDCl_3_) spectrum of Compound (**5m**), Figure S27. ^1^H-^1^HCOSY (CDCl_3_) spectrum of Compound (**5a)**, Figure S28. ^1^H-^1^H NOESY(CDCl_3_) spectrum of Compound (**5a)**, Figure S29. HMBC (CDCl_3_) spectrum of Compound (**5a)**, Figure S30. IR Spectra of Compound (**5a**). Table S1: MM-GBSA binding free energies components for the 1JIJ-5d Complex obtained from molecular dynamics trajectories; Table S2: MM-GBSA binding free energies components for the 2HCK-5d Complex obtained from molecular dynamics trajectories; Table S3: MM-GBSA binding free energies components for the 2QV4-5d Complex obtained from molecular dynamics trajectories.

## Sample Availability

Samples of the compounds are available from the authors.
