# Supplementary material for: Multifunctional Derivatives of Spiropyrrolidine Tethered Indeno-Quinoxaline Heterocyclic Hybrids as Potent Antimicrobial, Antioxidant and Antidiabetic Agents: Design, Synthesis, In Vitro and In Silico Approaches
Source: Molecules. 2022 Oct 25;27(21):7248. doi: 10.3390/molecules27217248 (PMC9653804; doi:10.3390/molecules27217248)
Supplement: Supplementary file 1 [file molecules-27-07248-s001.zip › molecules-1933901-supplementary.pdf]

Supplementary Information

Design and synthesis of novel class of spiropyrrolidine tethered indeno−quinoxaline heterocyclic hybrids as potent antimicrobial, antioxidant and antidiabetic agents. *In Vitro* and In Silico Studies

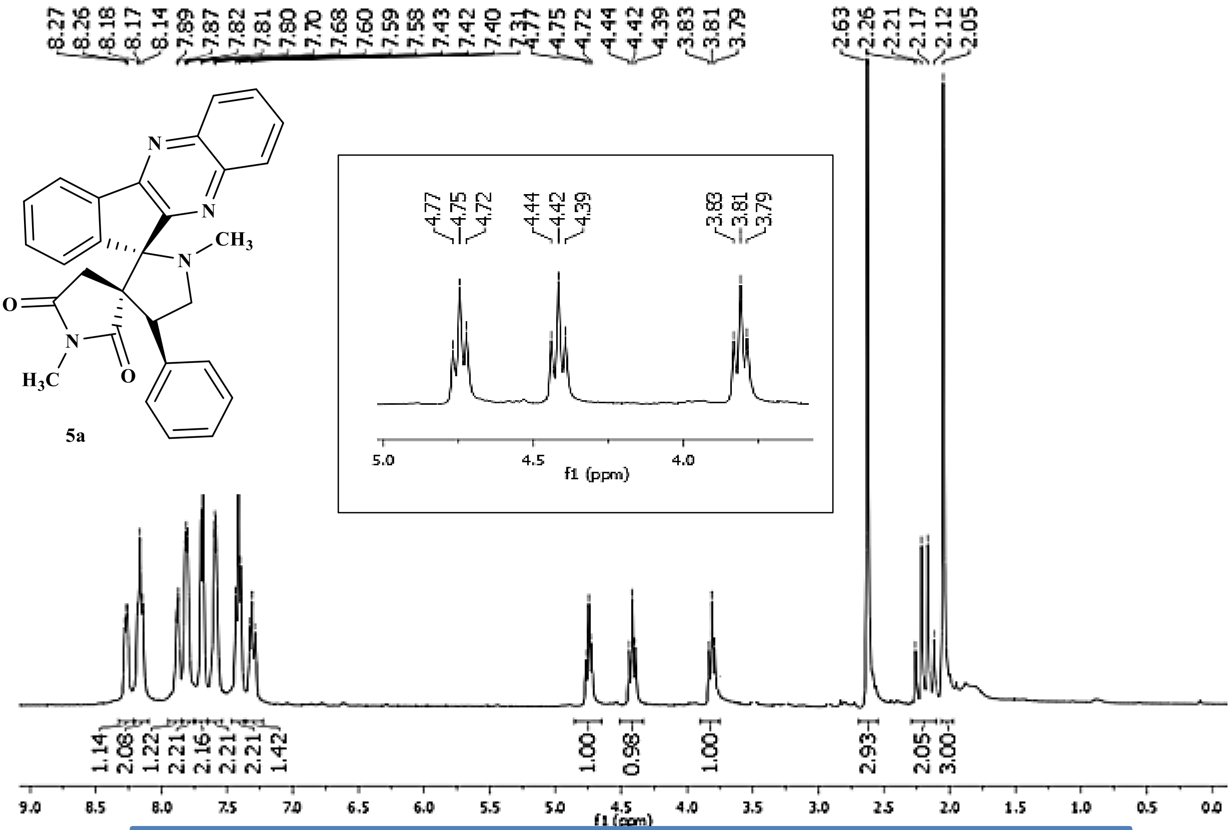

Figure S1. ^{1}H NMR ($CDCl_3$) spectrum of Compound (**5a**)

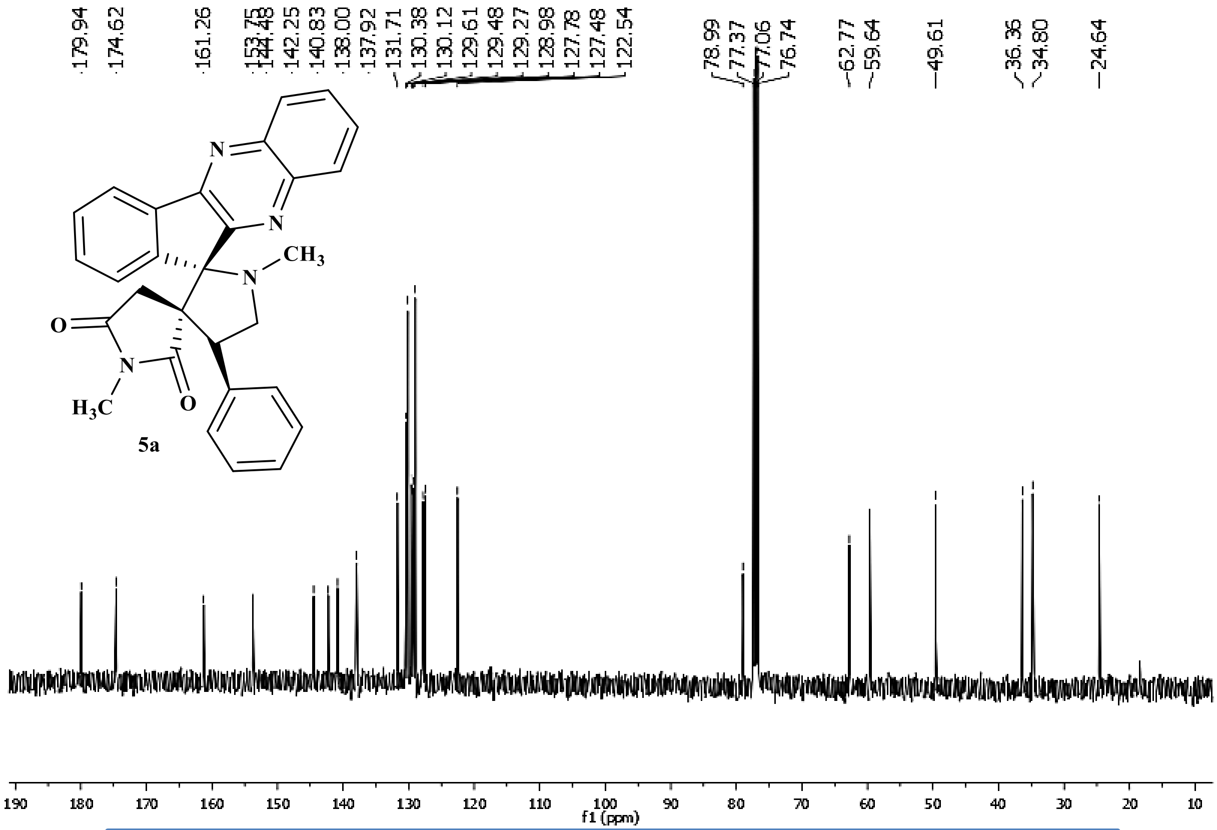

Figure S2. ^{13}C NMR ($CDCl_3$) spectrum of Compound (**5a**)

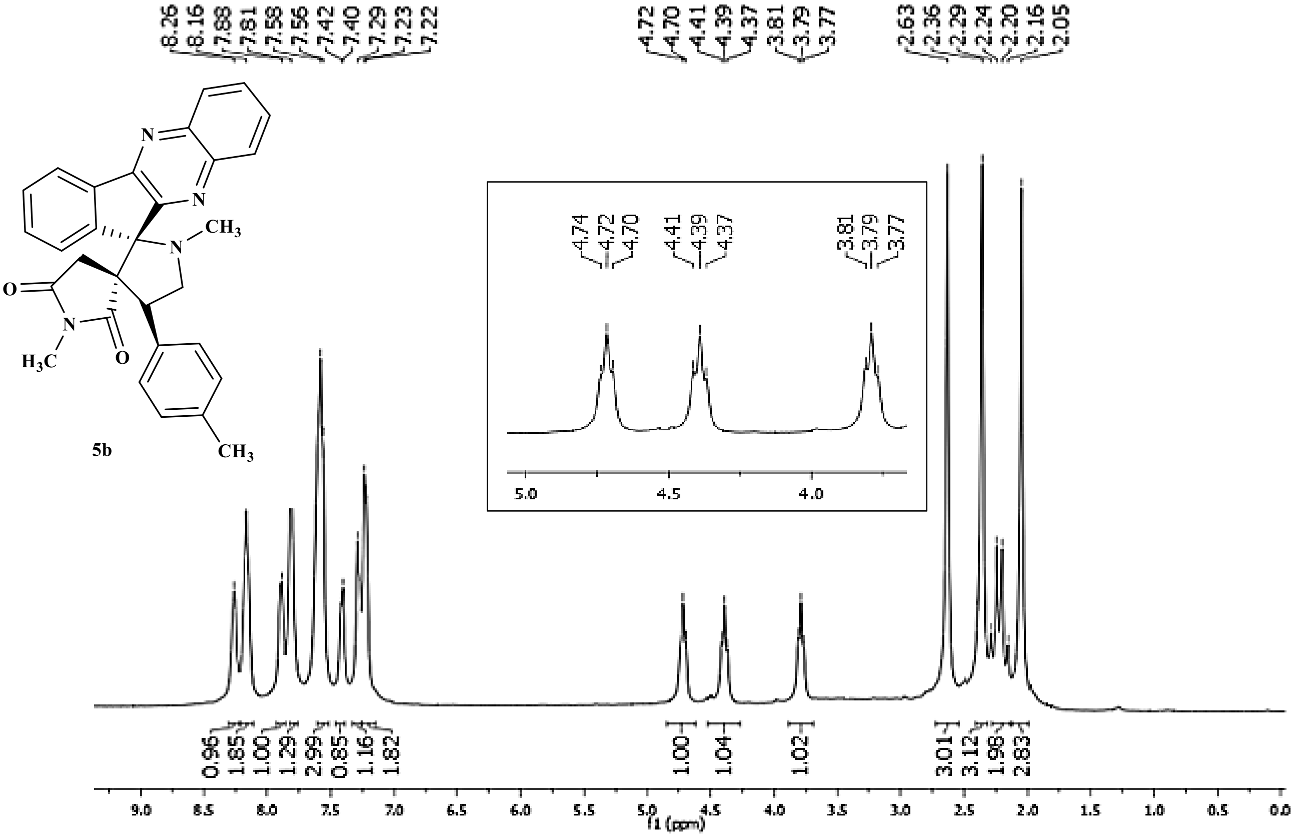

Figure S3. ^{1}H NMR (CDCl3) spectrum of Compound (**5b**)

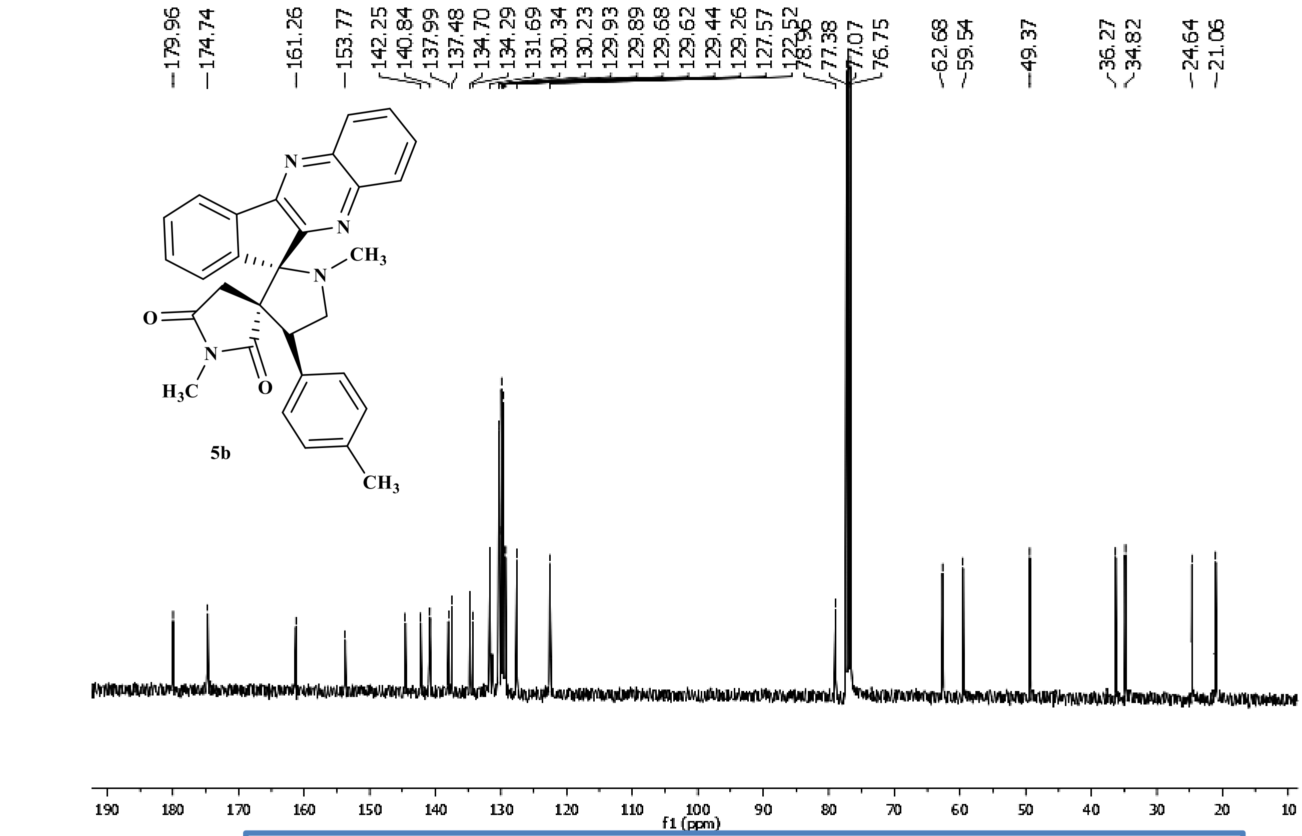

Figure S4. ^{13}C NMR (CDCl3) spectrum of Compound (**5b**)

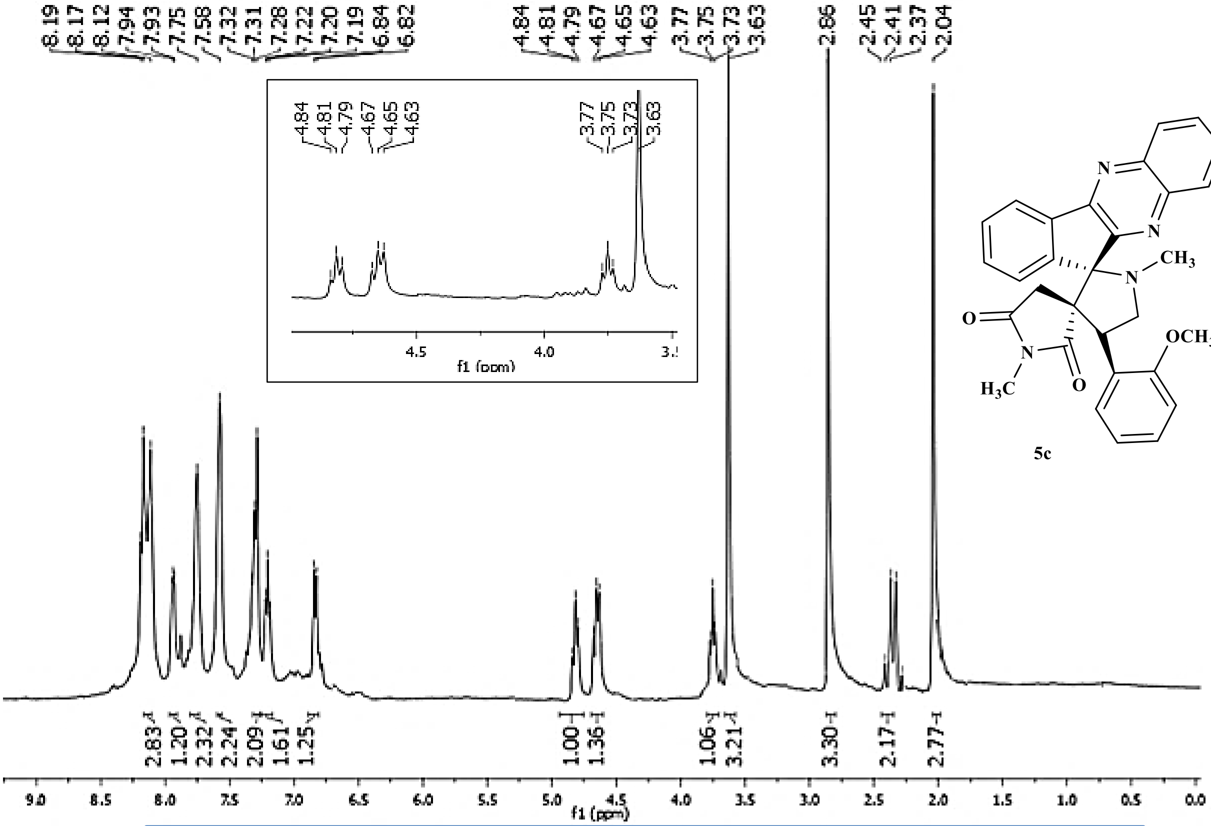

Figure S5. ^{1}H NMR (CDCl3) spectrum of Compound (**5c**)

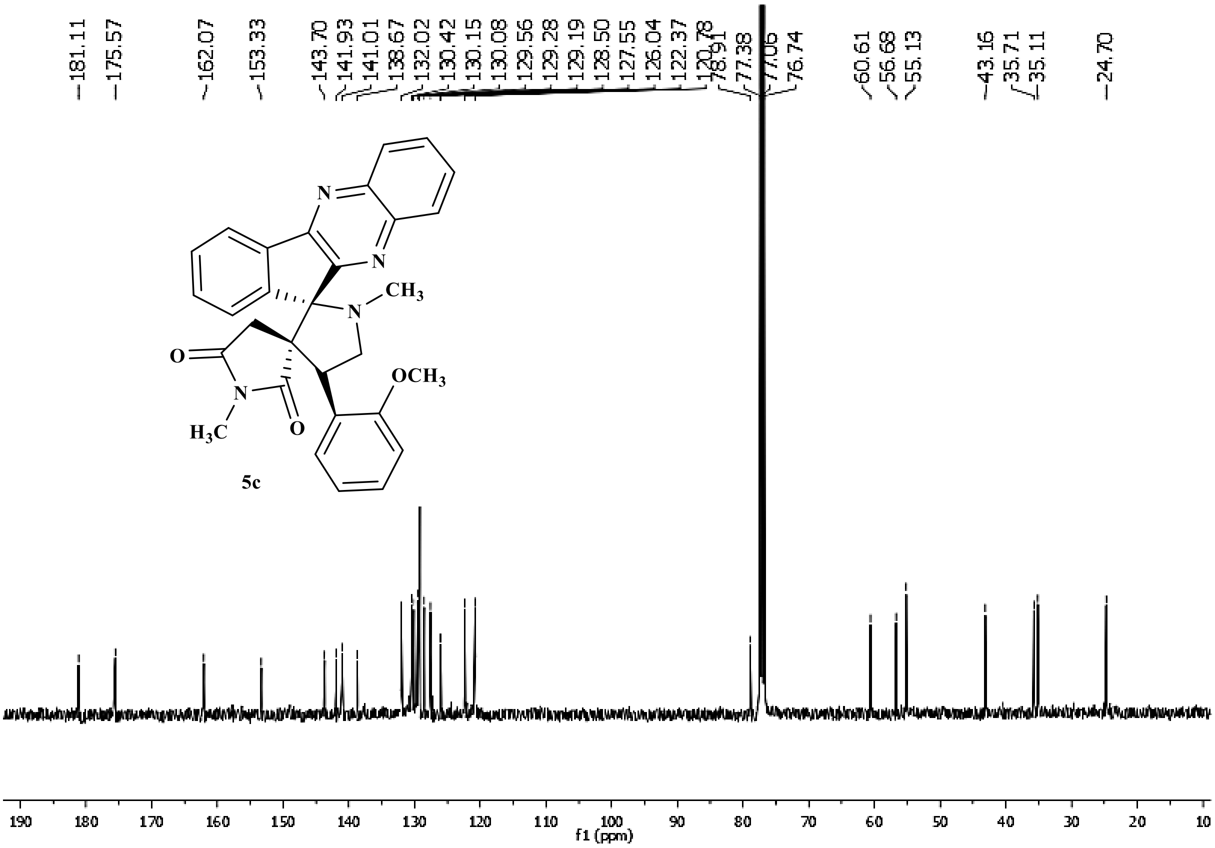

Figure S6. ^{13}C NMR (CDCl3) spectrum of Compound (**5c**)

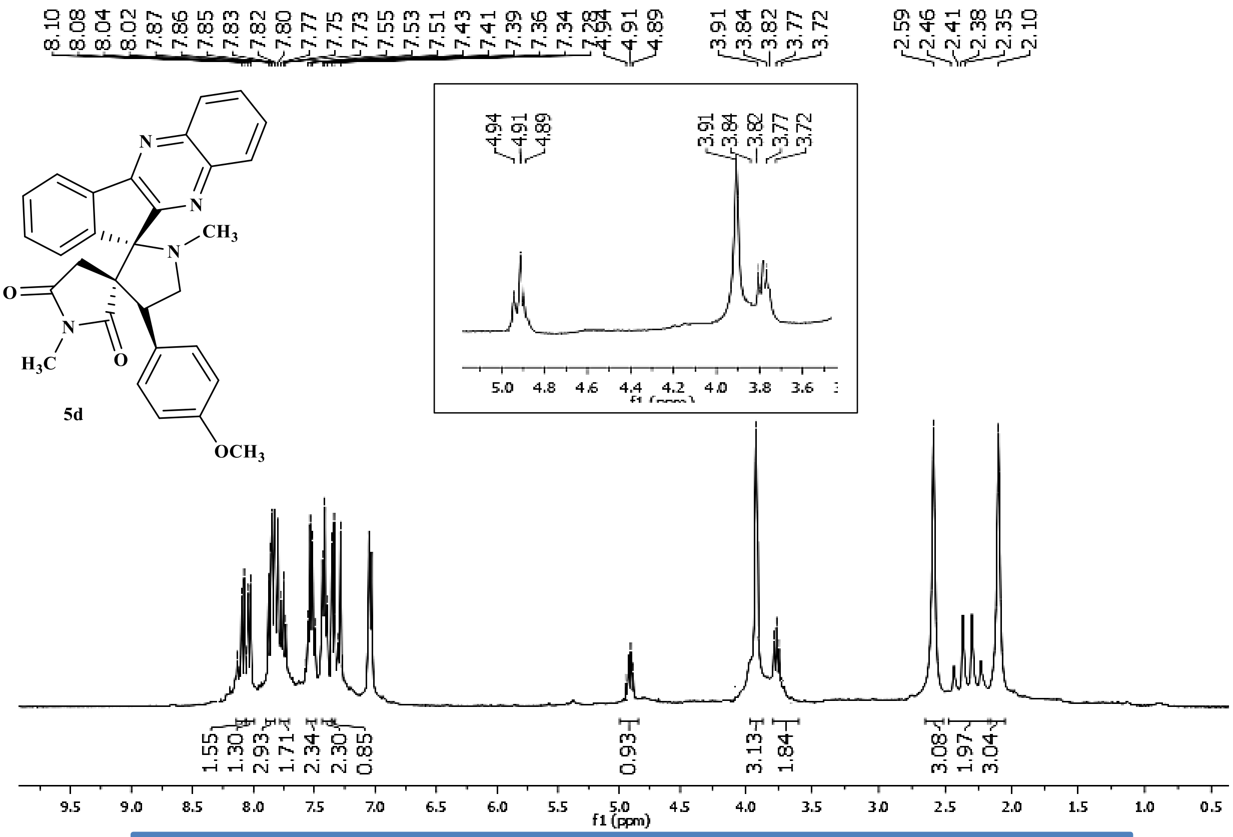

Figure S7. ^{1}H NMR (CDCl3) spectrum of Compound (**5d**)

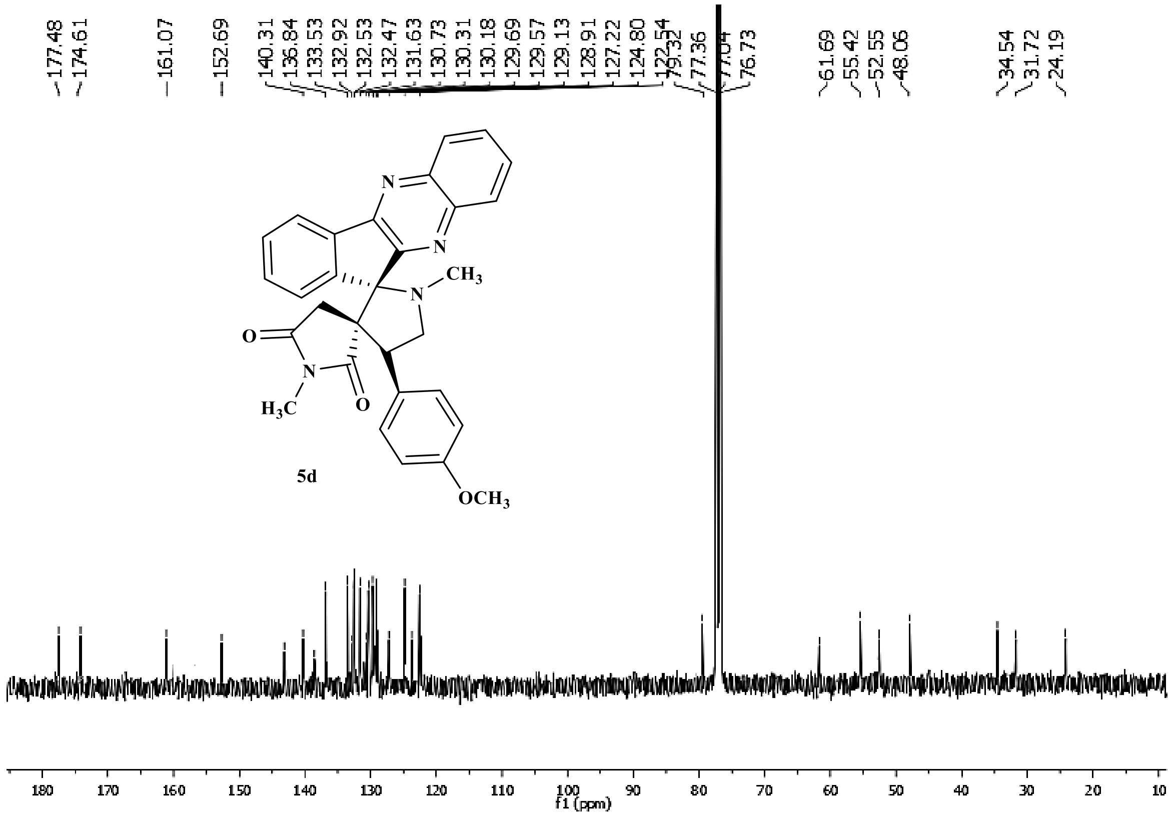

Figure S8. ^{13}C NMR (CDCl3) spectrum of Compound (**5d**)

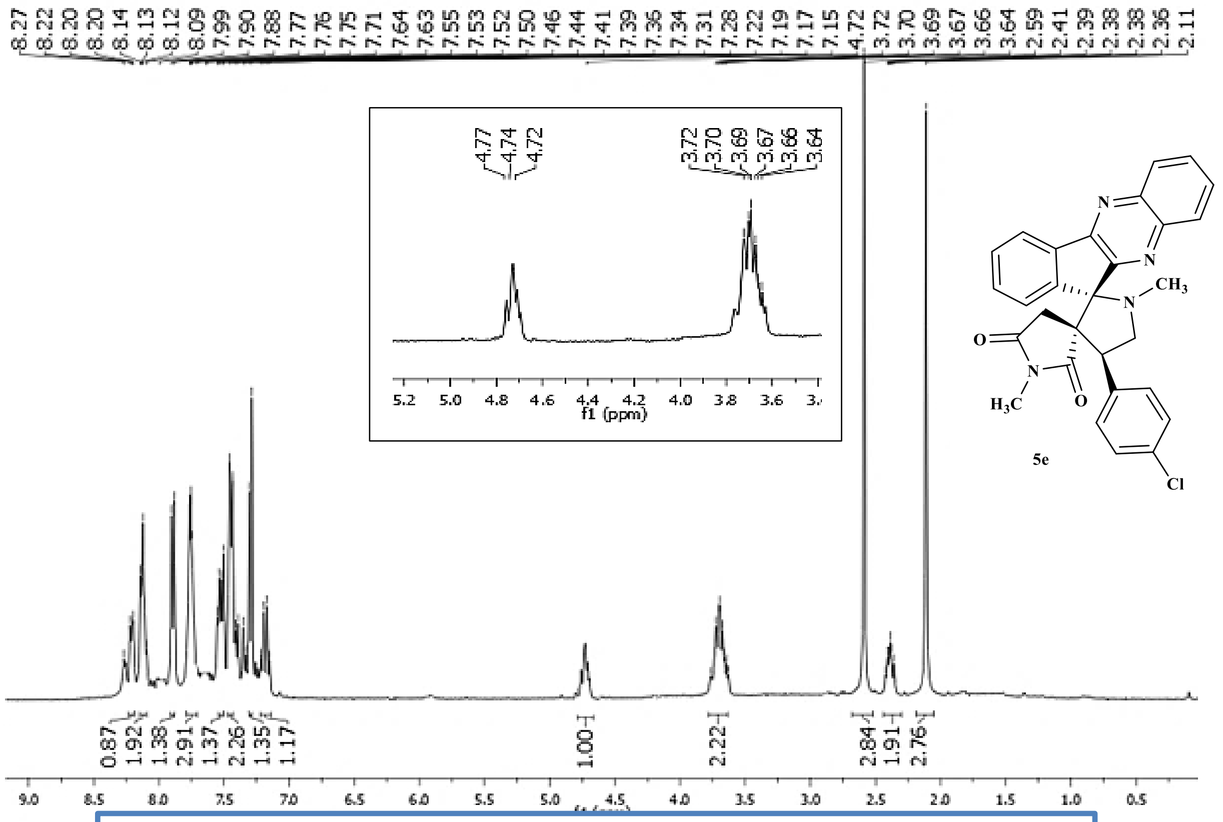

Figure S9. ^{1}H NMR ($CDCl_3$) spectrum of Compound (**5e**)

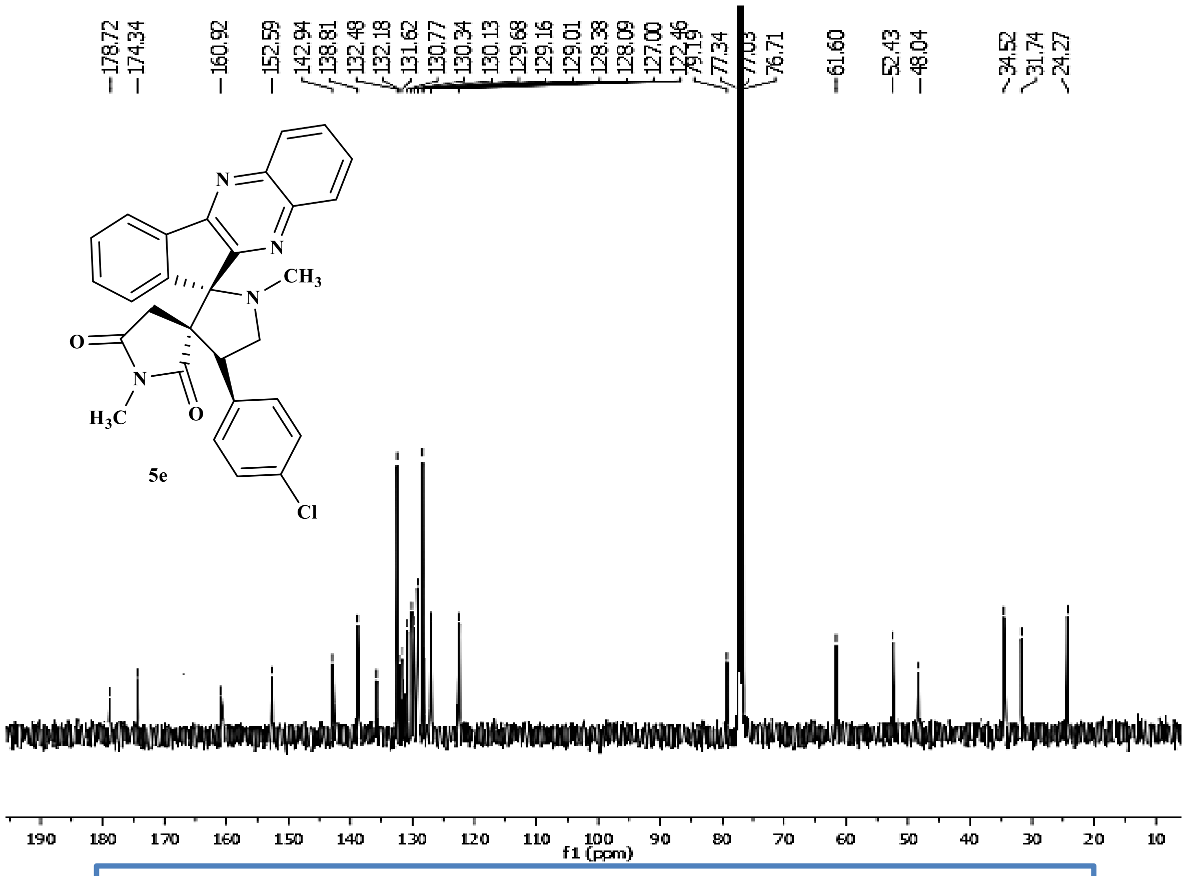

Figure S10. ^{13}C NMR ($CDCl_3$) spectrum of Compound (**5e**)

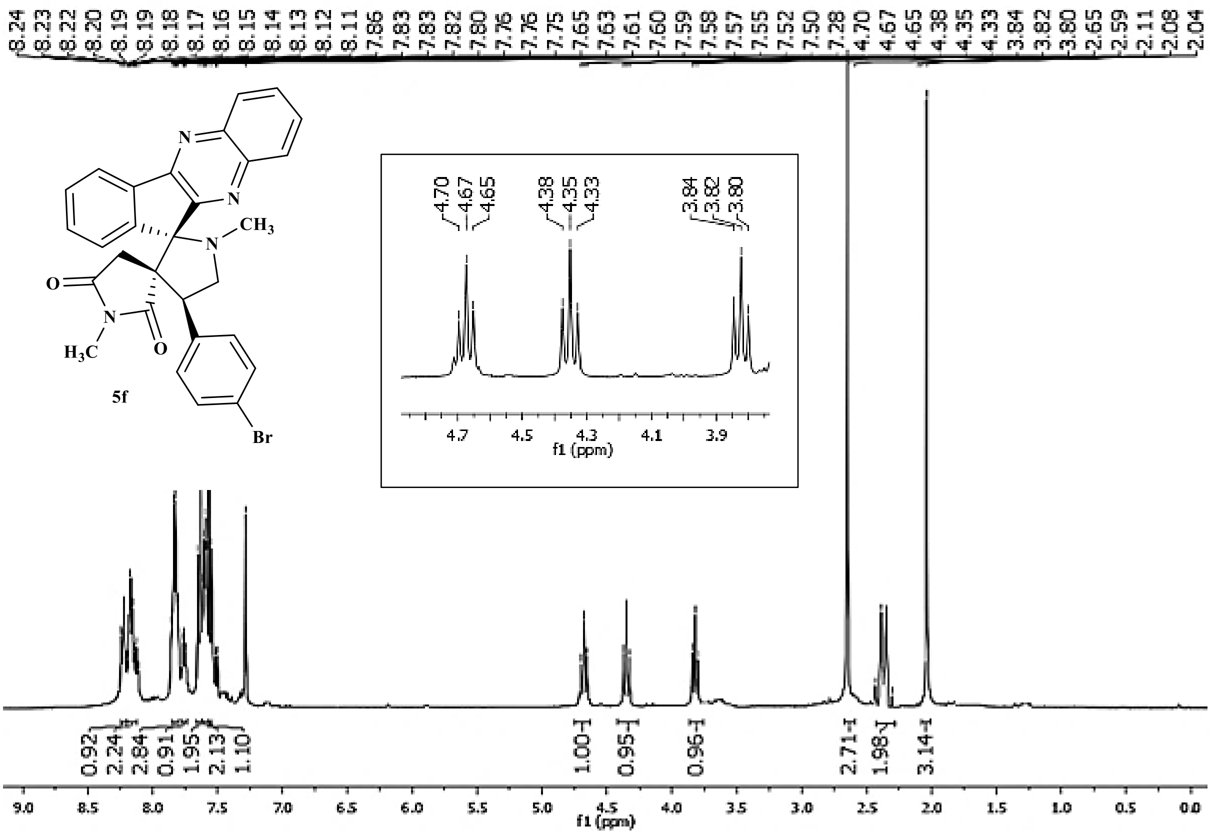

Figure S11. ^{1}H NMR (CDCl3) spectrum of Compound (**5f**)

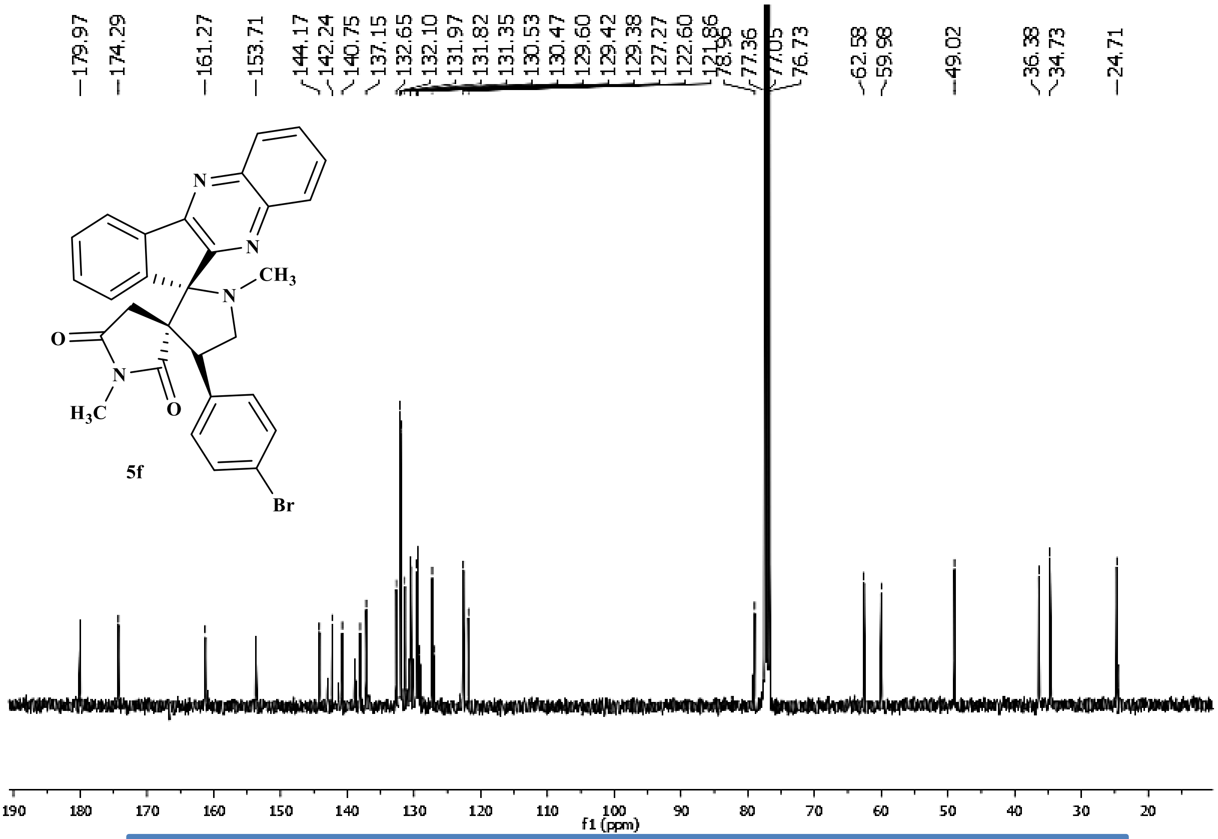

Figure S12. ^{13}C NMR (CDCl3) spectrum of Compound (**5f**)

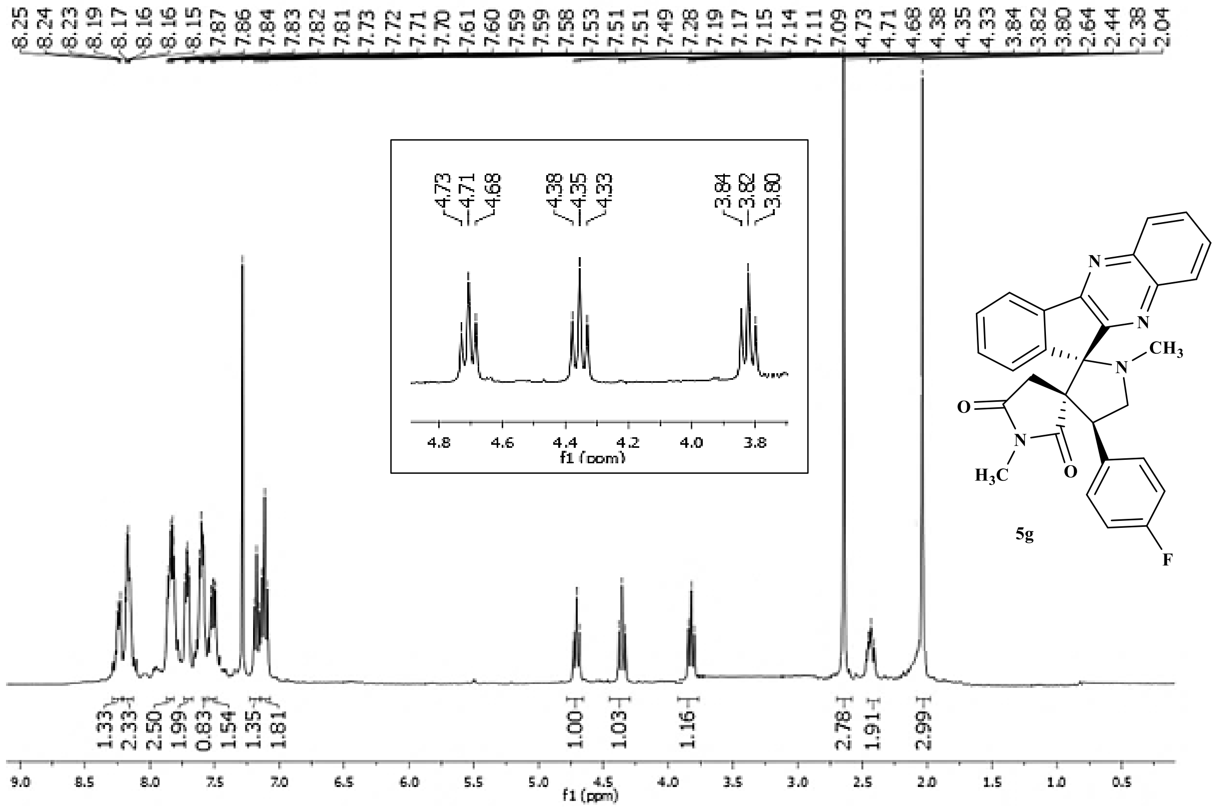

Figure S13. ^{1}H NMR (CDCl3) spectrum of Compound (**5g**)

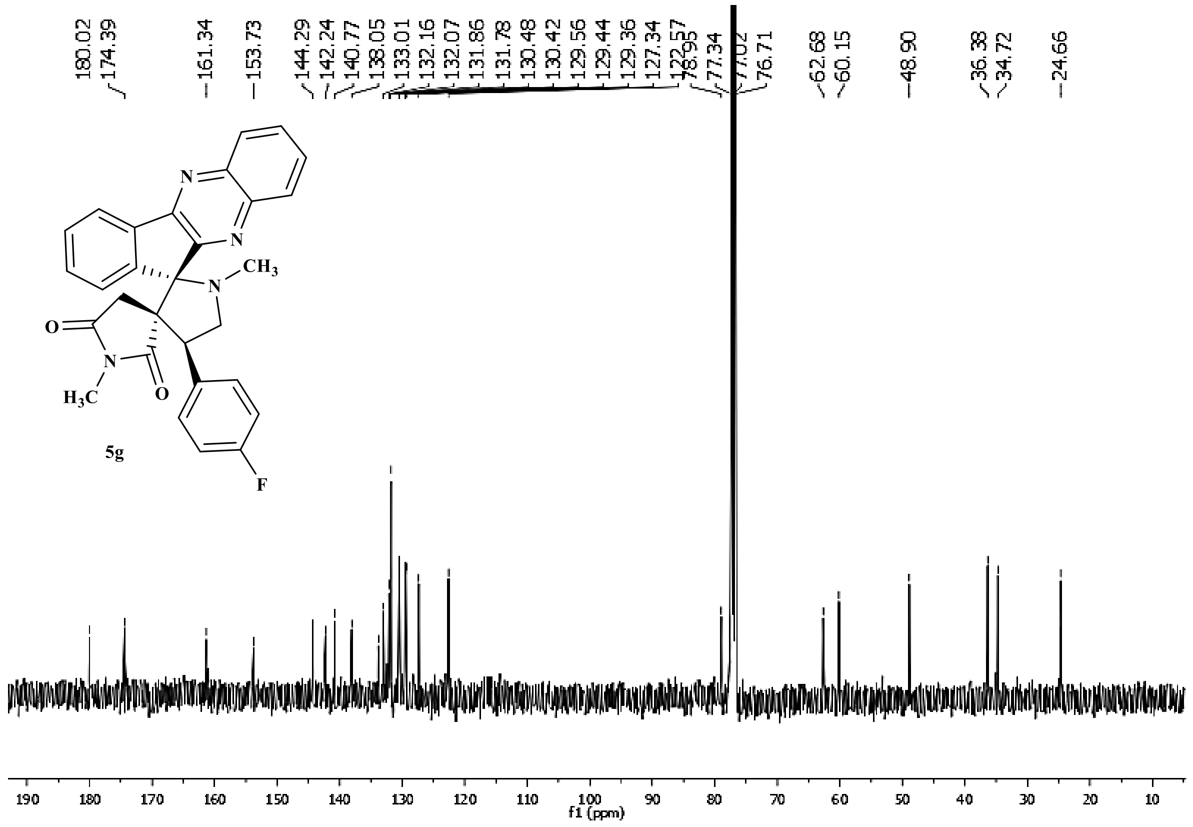

Figure S14. ^{13}C NMR (CDCl3) spectrum of Compound (**5g**)

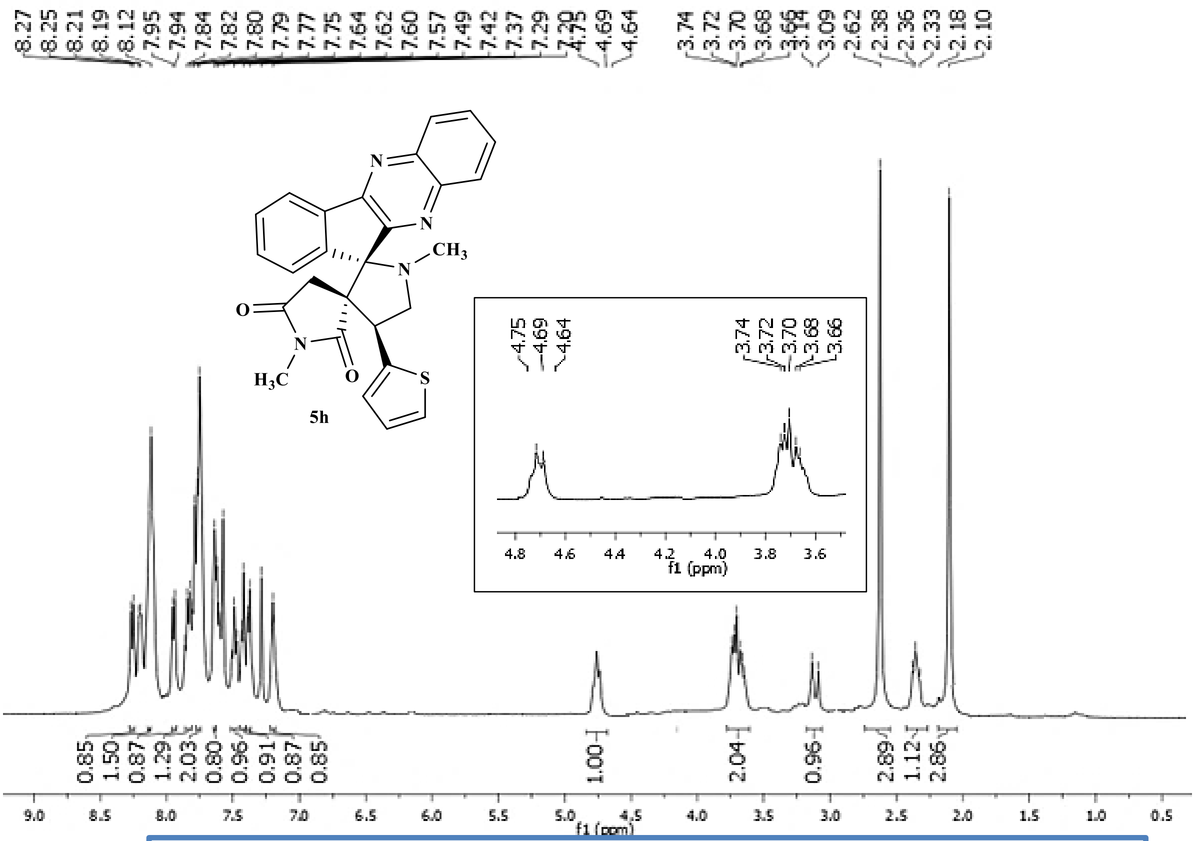

Figure S15. ^{1}H NMR ($CDCl_3$) spectrum of Compound (**5h**)

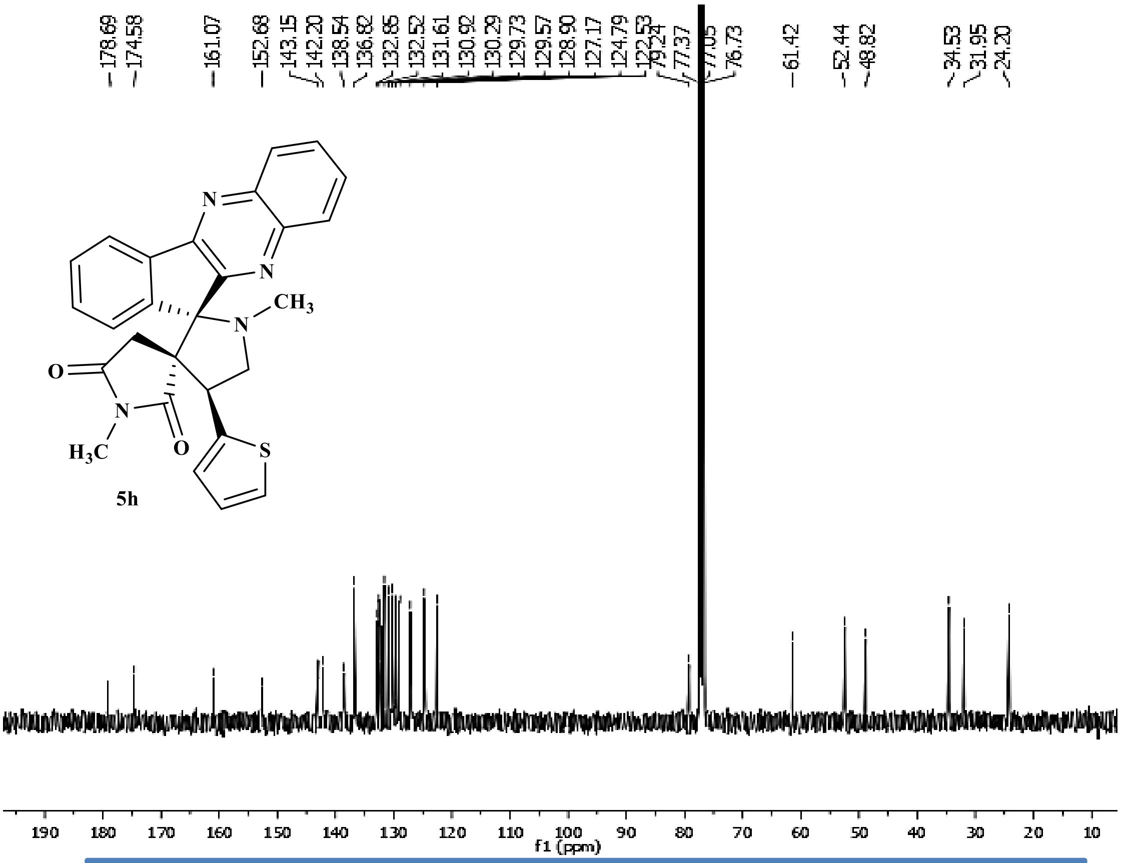

Figure S16. ^{13}C NMR ($CDCl_3$) spectrum of Compound (**5h**)

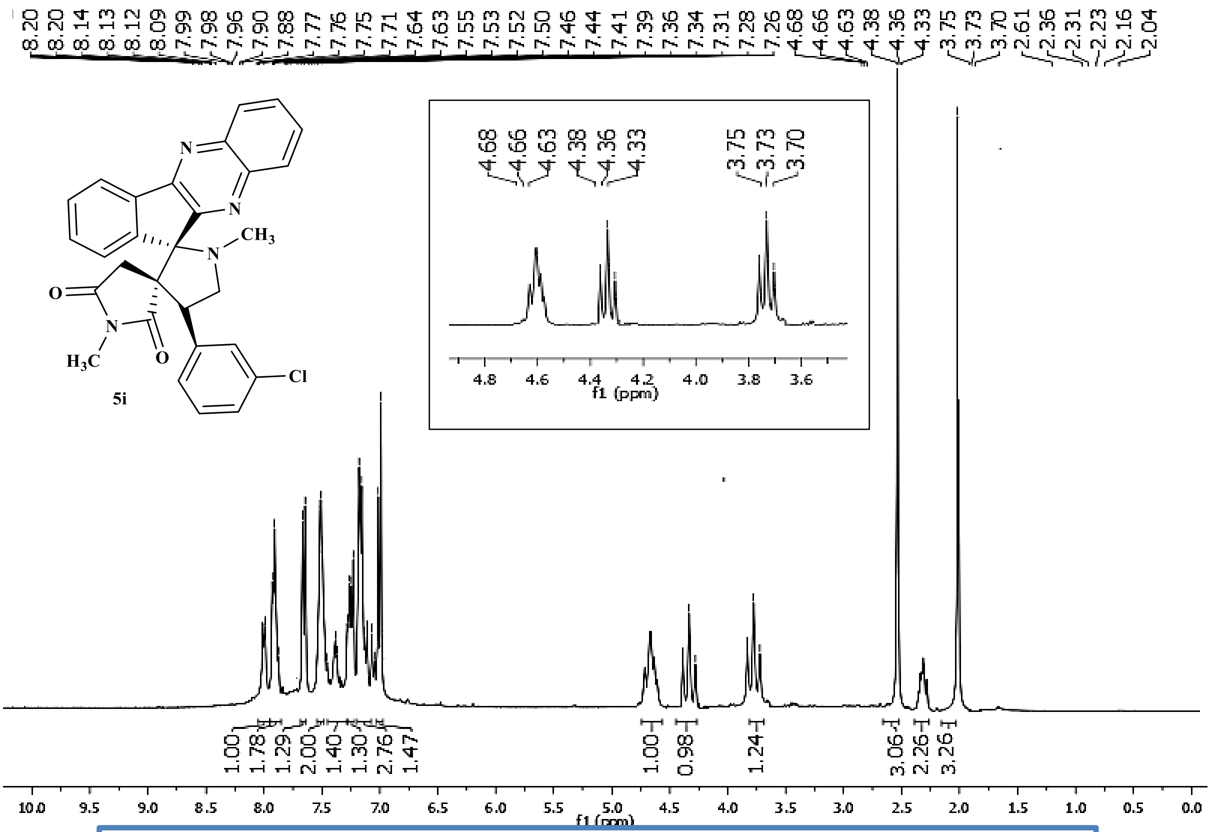

Figure S17. ^{1}H NMR ($CDCl_3$) spectrum of Compound (**5i**)

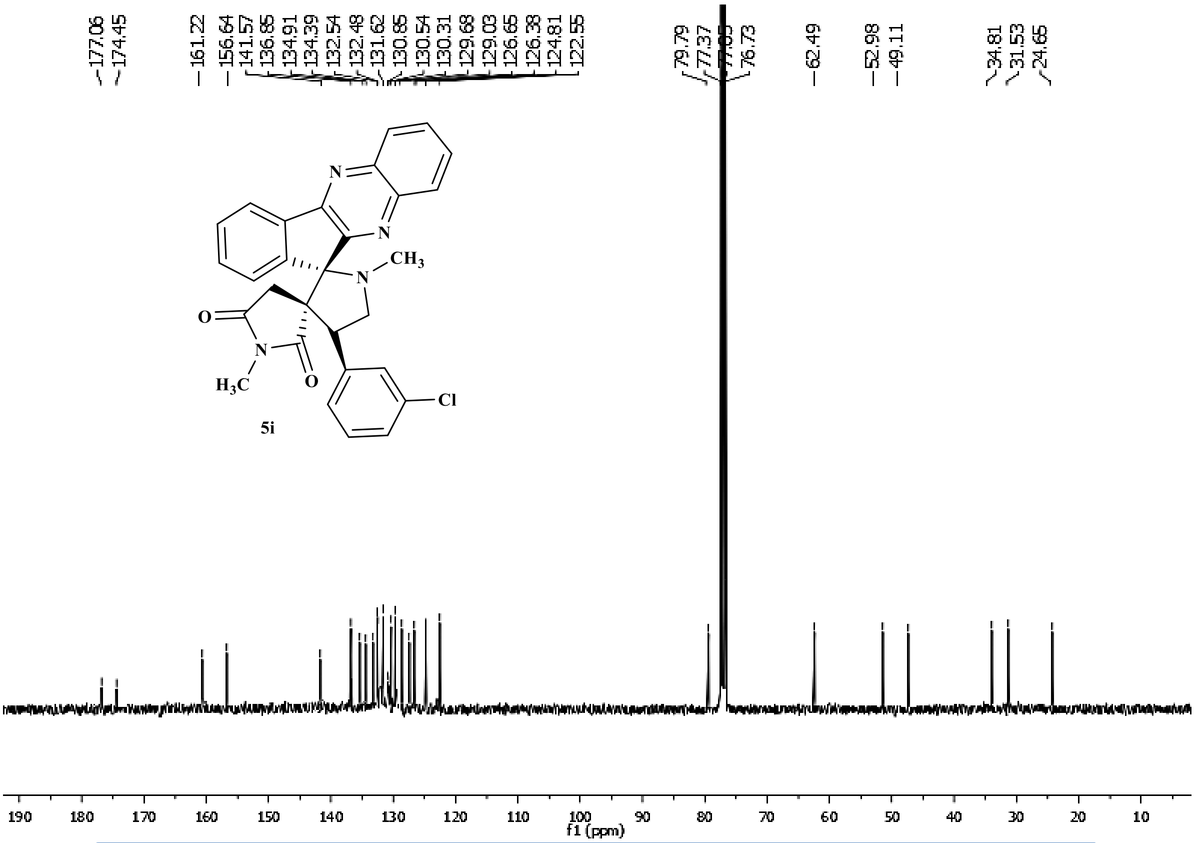

Figure S18. ^{13}C NMR ($CDCl_3$) spectrum of Compound (**5i**)

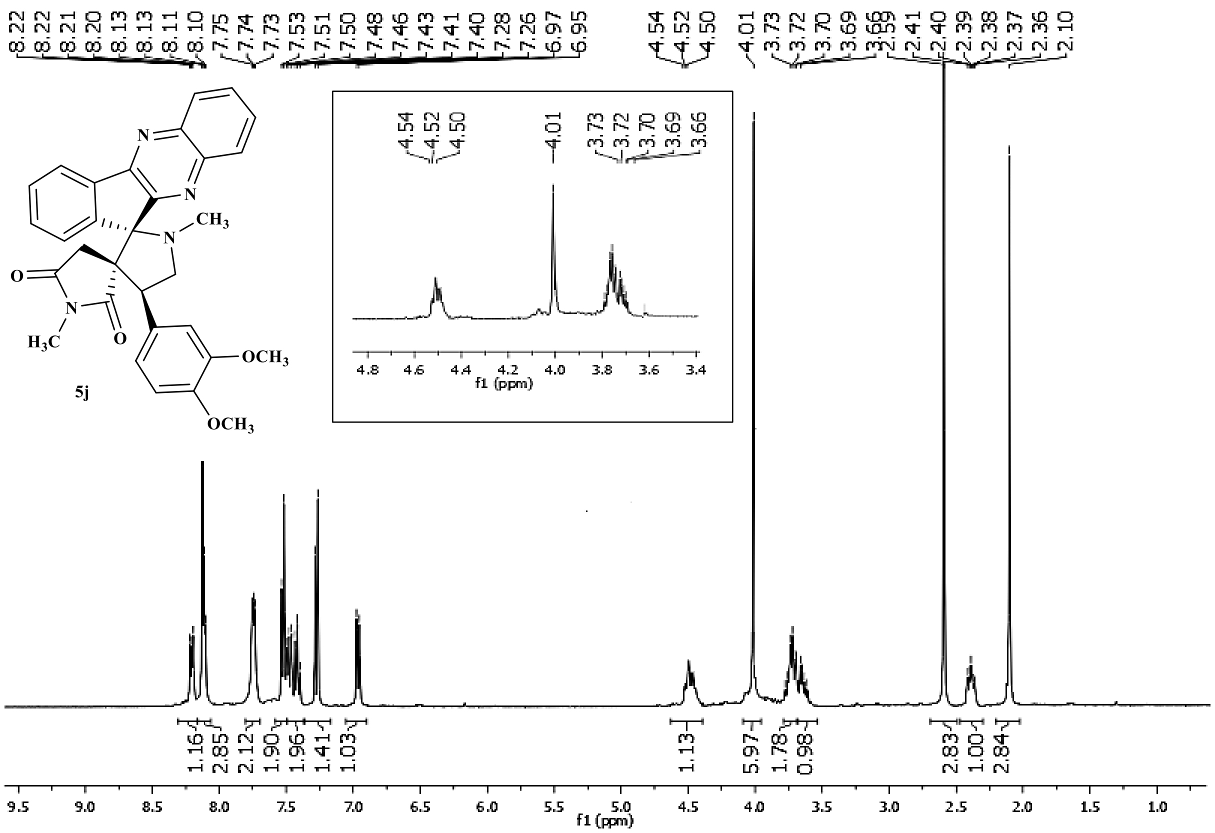

Figure S19. ^{1}H NMR ($CDCl_3$) spectrum of Compound (**5j**)

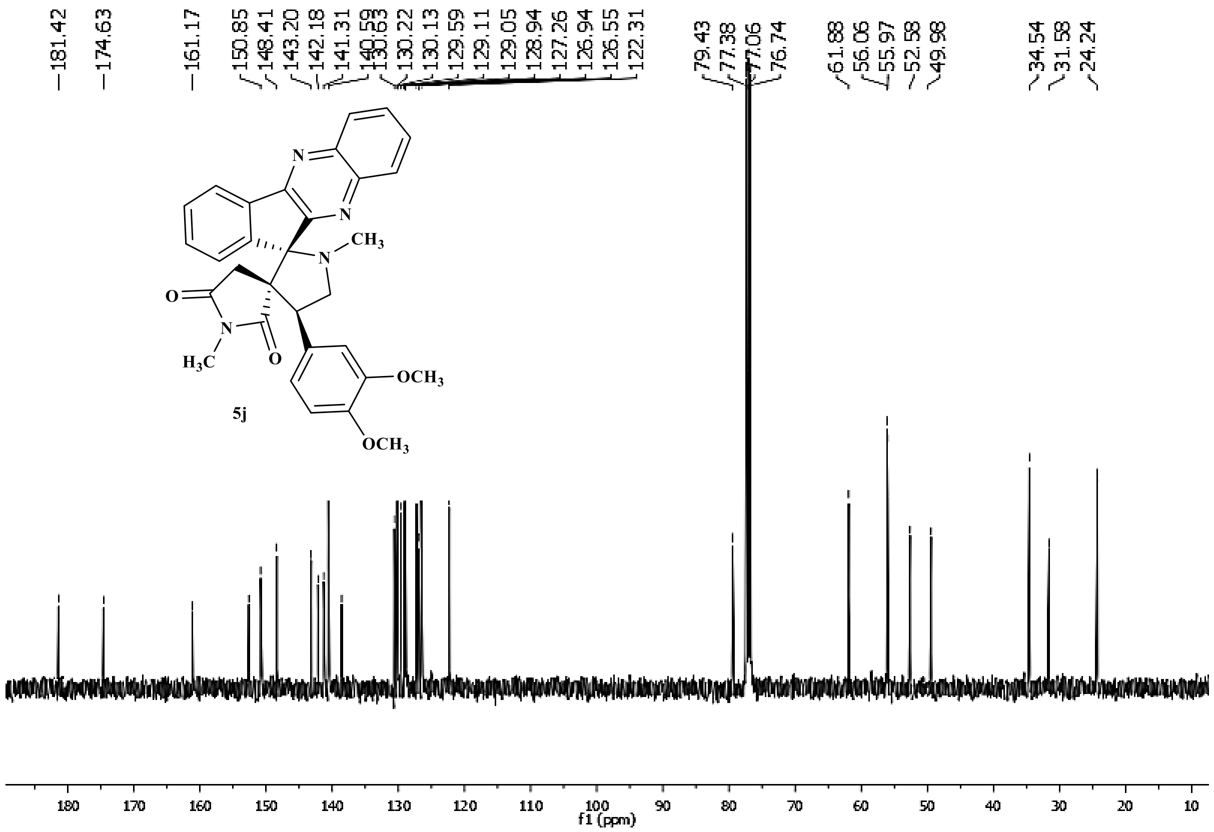

Figure S20. ^{13}C NMR ($CDCl_3$) spectrum of Compound (**5j**)

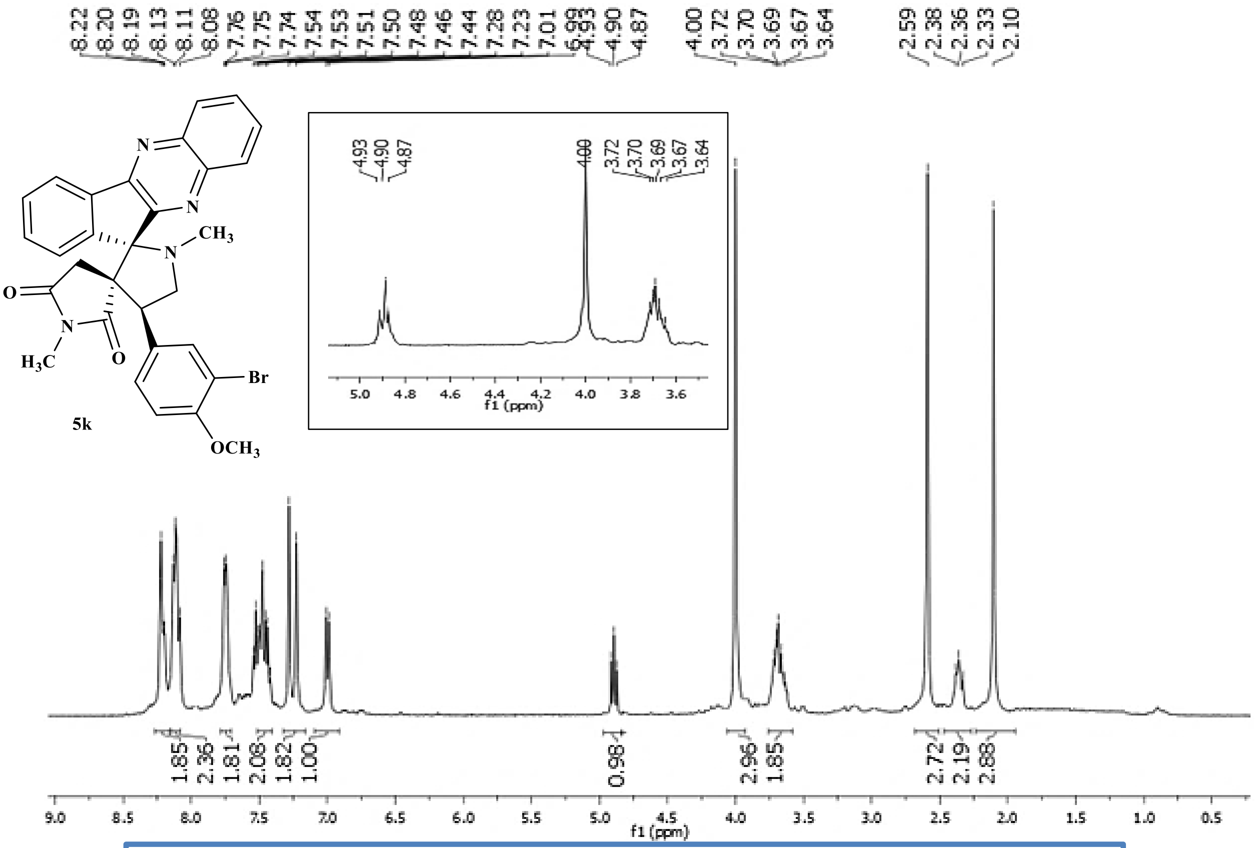

Figure S21. ^{1}H NMR ($CDCl_3$) spectrum of Compound (**5k**)

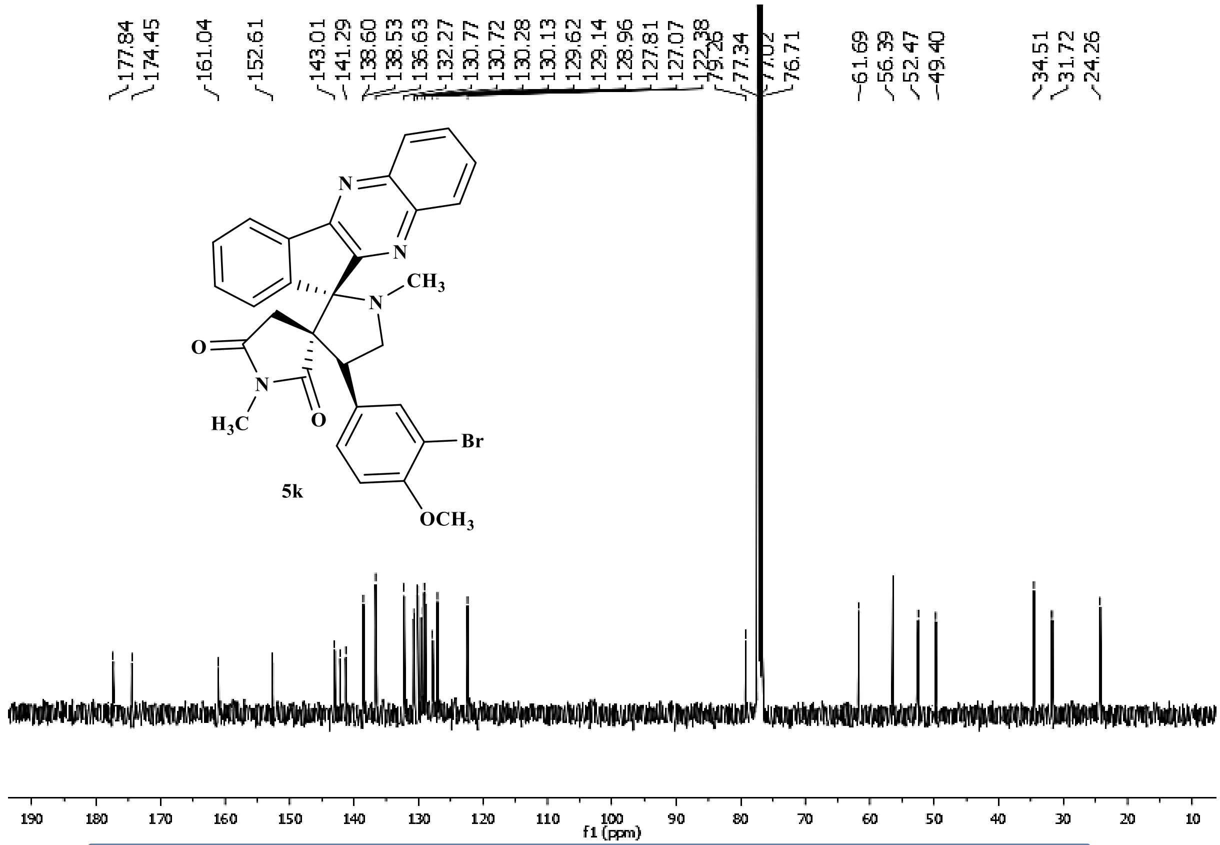

Figure S22. ^{13}C NMR ($CDCl_3$) spectrum of Compound (**5k**)

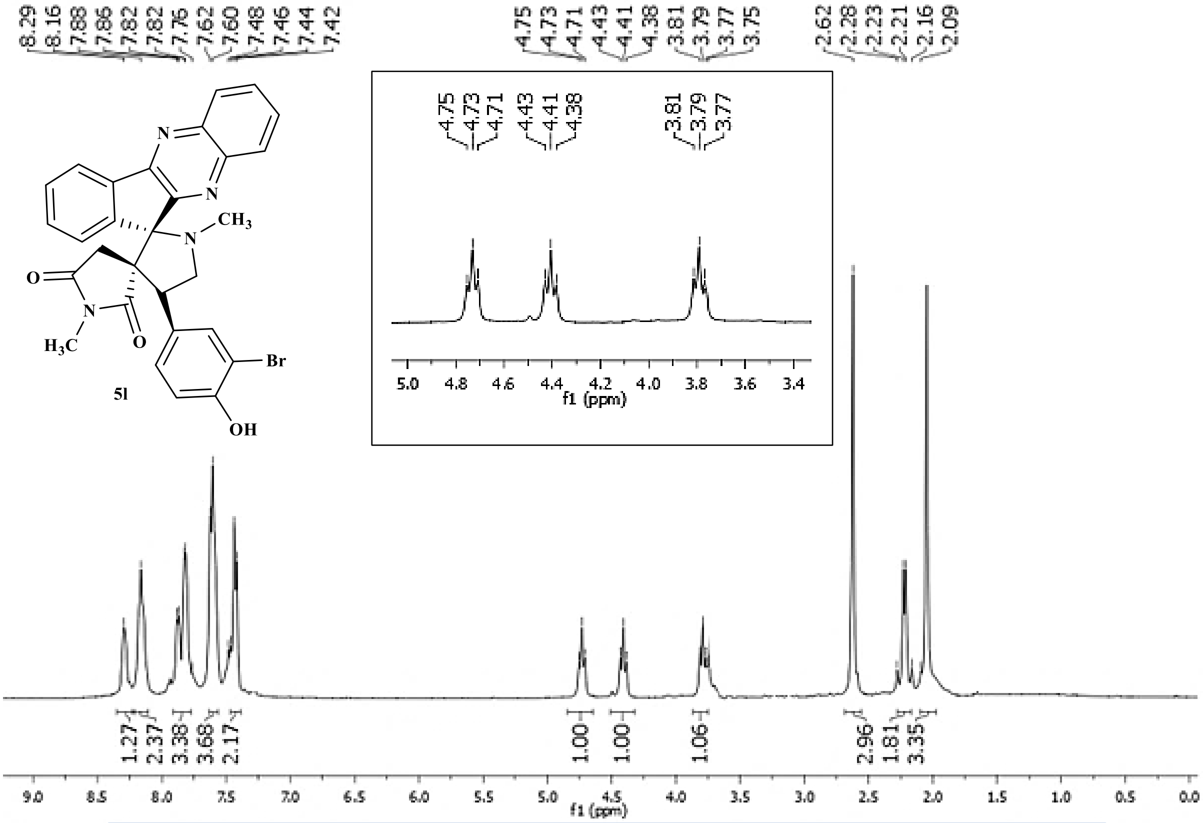

Figure S23. ^{1}H NMR (CDCl3) spectrum of Compound (**5l**)

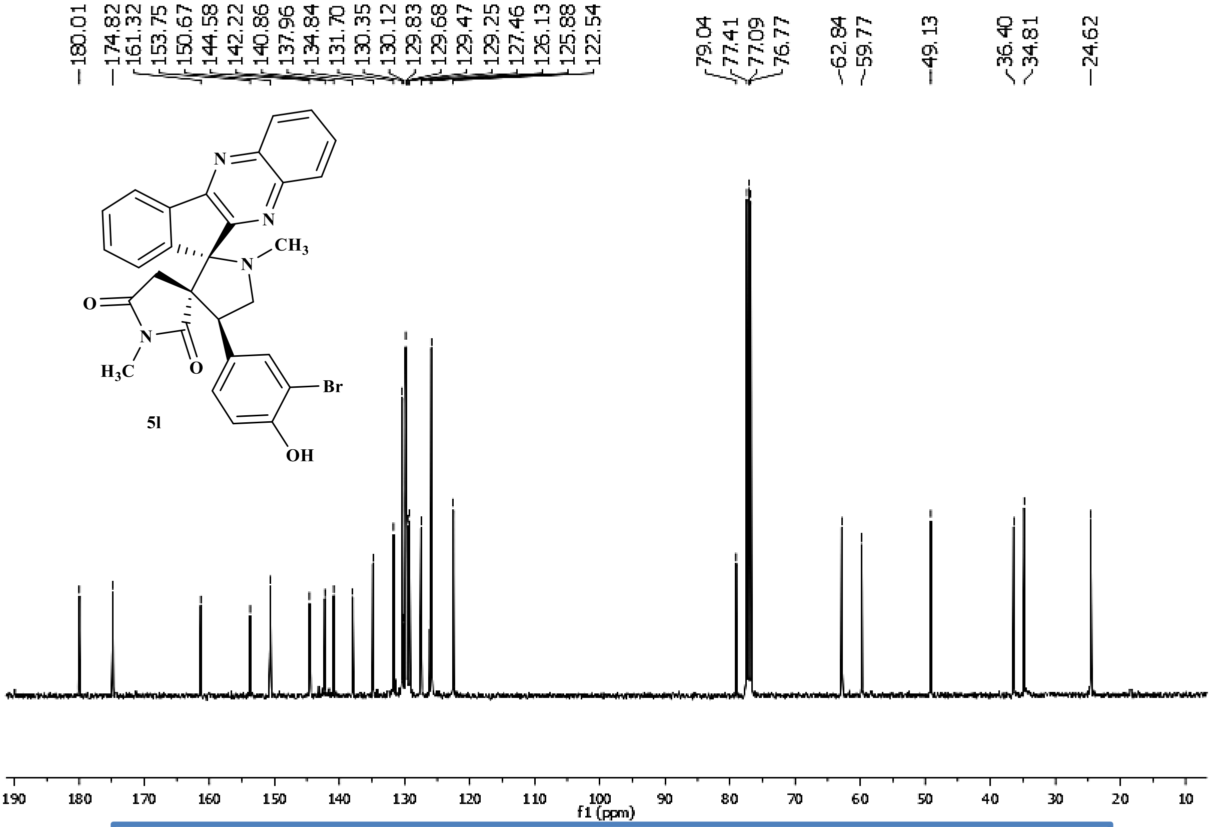

Figure S24. ^{13}C NMR (CDCl3) spectrum of Compound (**5l**)

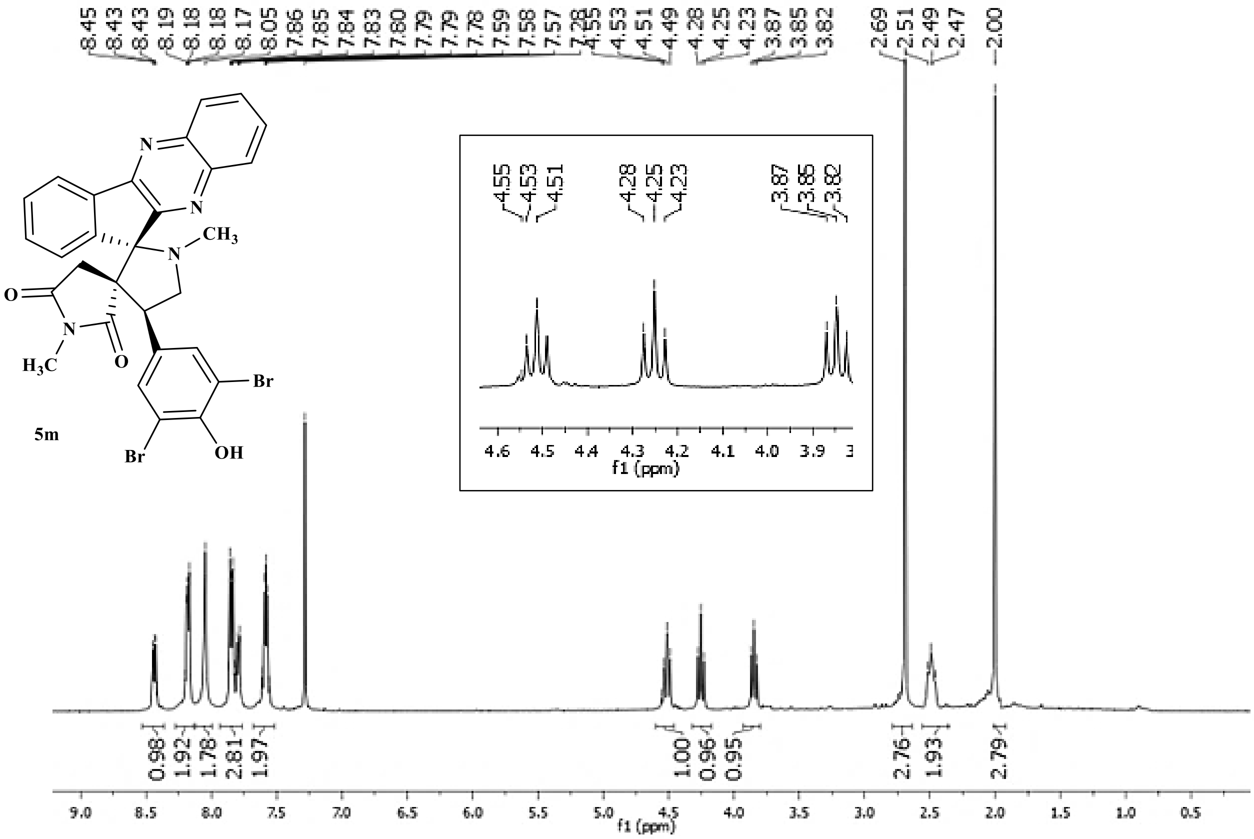

Figure S25. ^{1}H NMR (CDCl3) spectrum of compound (**5m**)

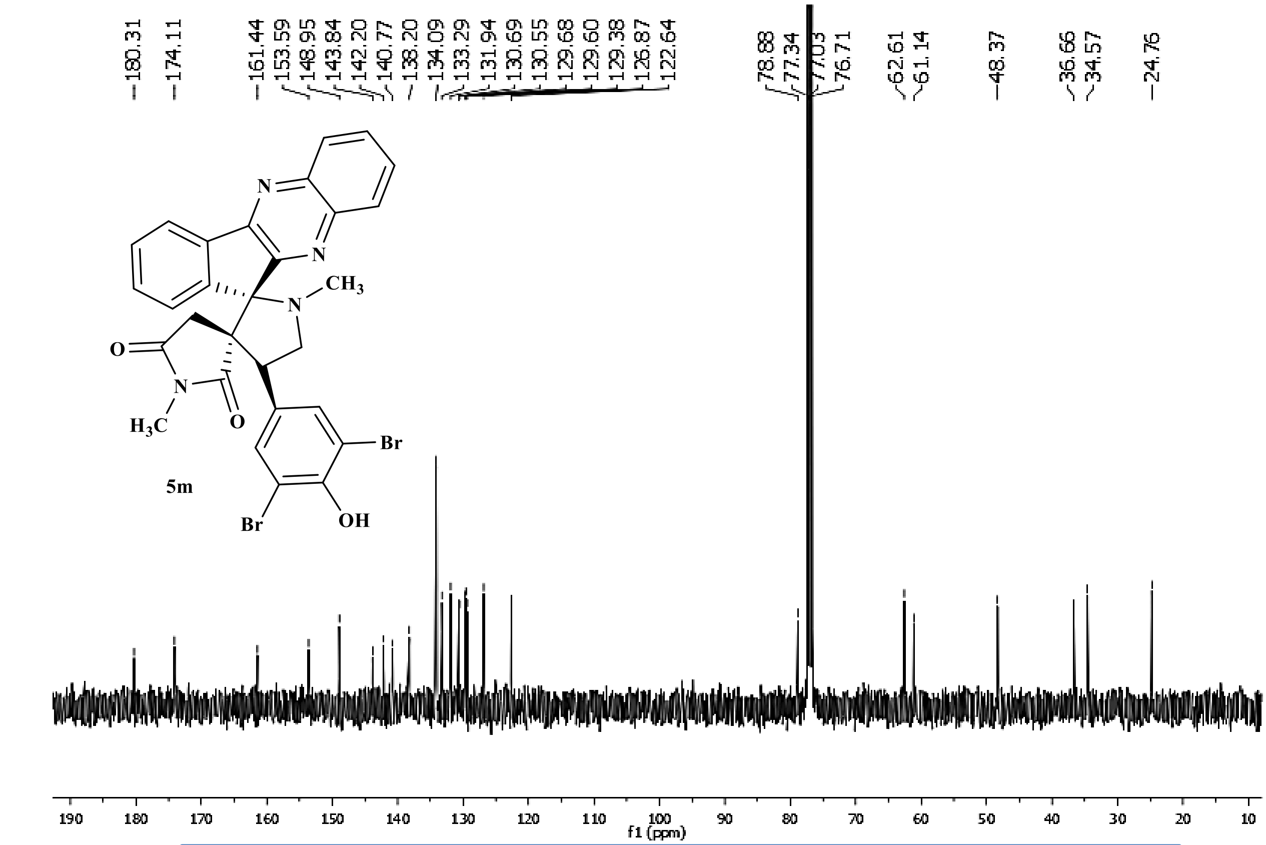

Figure S26. ^{13}C NMR (CDCl3) spectrum of Compound (**5m**)

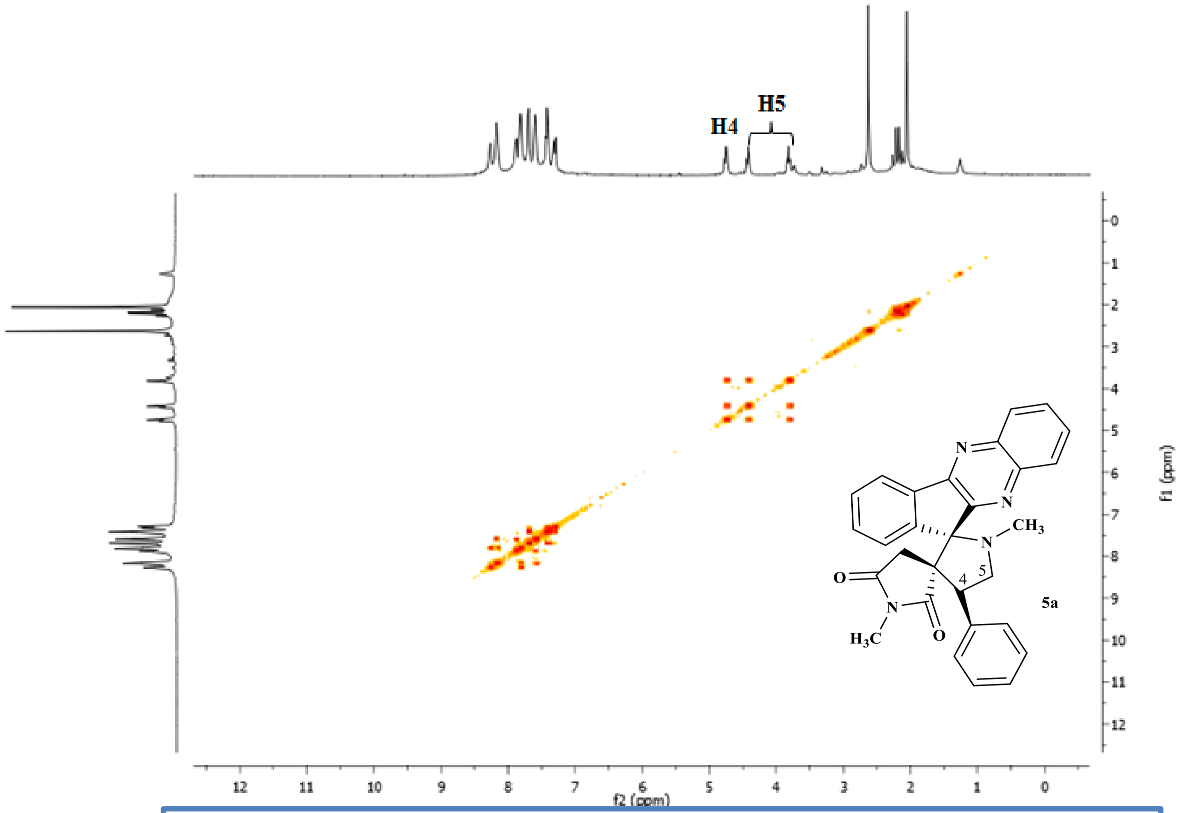

Figure S27. ^{1}H-^{1}HCOSY ($CDCl_3$) spectrum of Compound **5a**

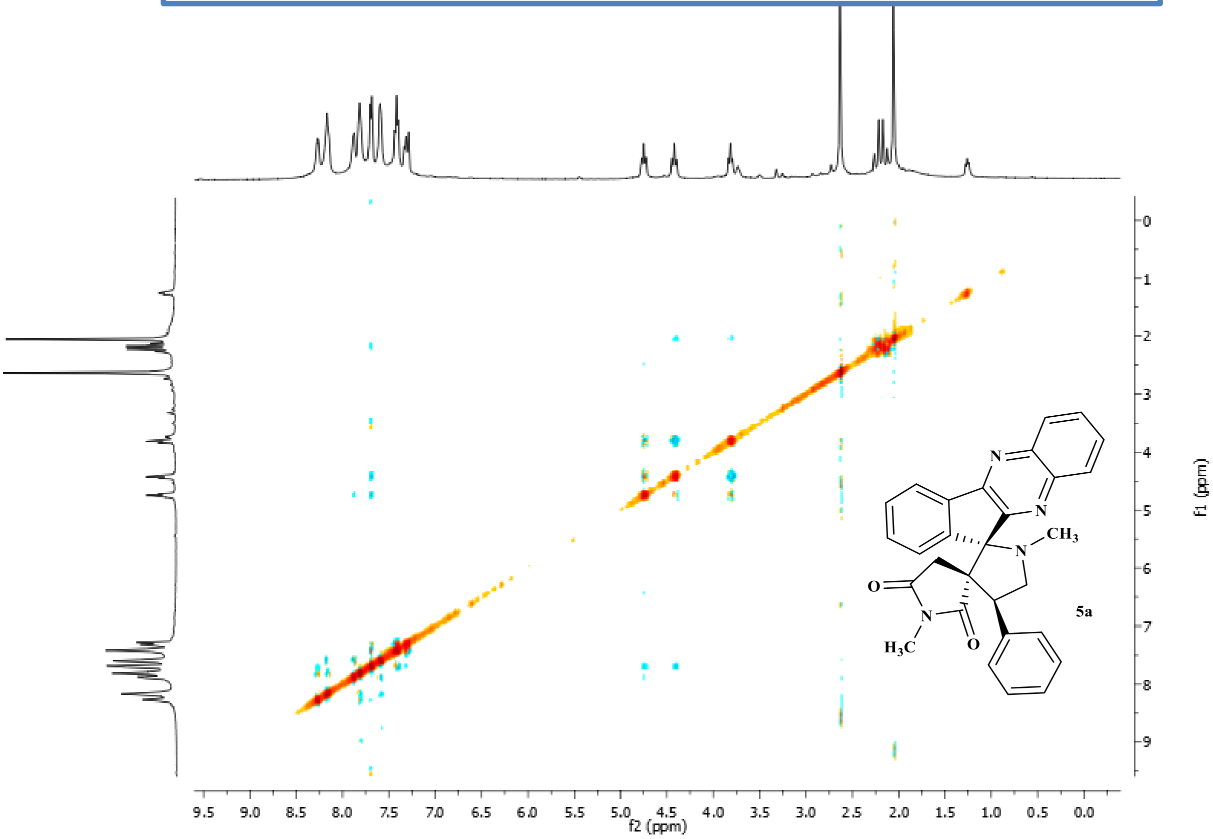

Figure S28. ^{1}H-^{1}H NOESY($CDCl_3$) spectrum of Compound **5a**

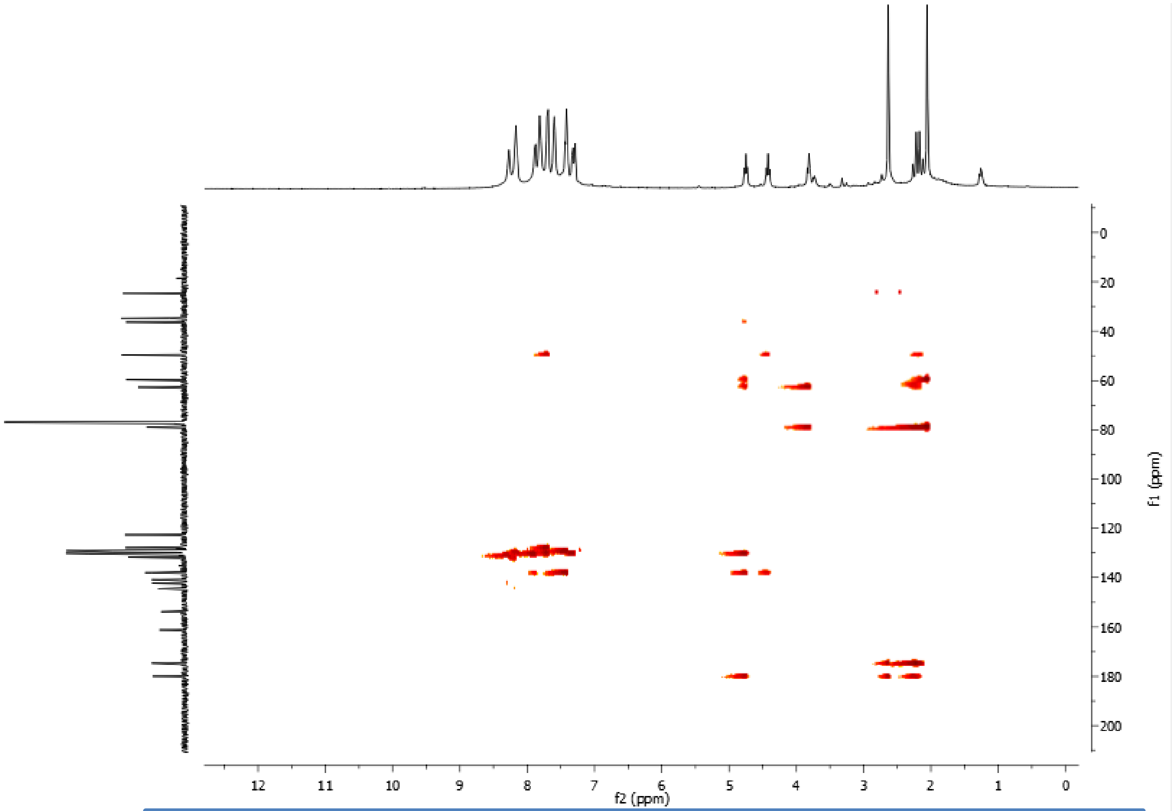

Figure S29. HMBC (CDCl3) spectrum of Compound **5a**

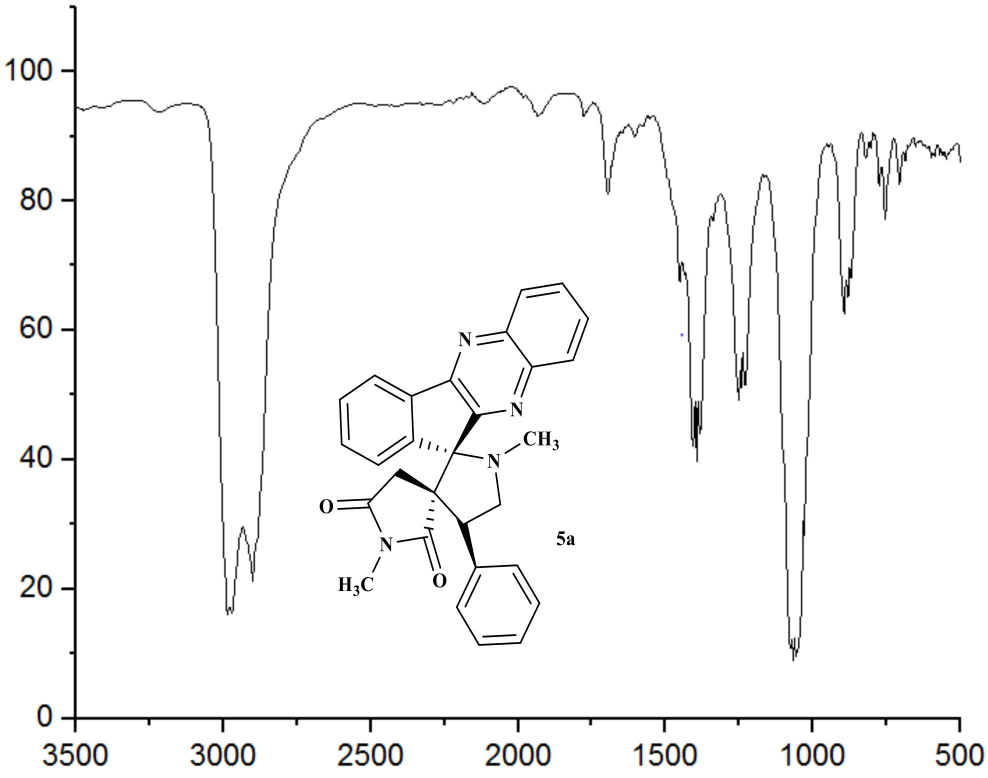

Figure S30. IR Spectra of Compound **5a**

Molecular docking and dynamic simulation part

Table S1: MM−GBSA binding free energies components for the 1JIJ−5d Complex obtained from molecular dynamics trajectories.

Frame No.	MMGBSA					
	ΔG Bind	ΔG Bind Coulomb	ΔG Bind H bond	ΔG Bind Lipo	ΔG Bind Solv GB	ΔG Bind VDW
0	−52.35	−8.66	−0.50	−19.00	32.50	−57.37
1	−54.04	−7.42	−0.98	−20.28	36.24	−60.68
2	−46.23	−7.62	−0.27	−16.52	33.82	−55.37
3	−53.83	−7.46	−0.84	−18.39	34.37	−60.90
4	−47.31	−7.73	−0.87	−17.31	34.07	−55.91
5	−53.73	−7.09	−0.76	−19.21	34.00	−60.13
6	−44.84	−14.75	−0.87	−15.82	33.76	−48.92
7	−49.19	−9.96	−0.85	−17.56	35.16	−55.64
8	−48.08	−11.35	−1.05	−17.54	40.50	−59.87
9	−49.79	−8.43	−0.89	−17.89	36.05	−59.40
10	−52.84	−8.10	−1.00	−17.85	33.73	−58.90
11	−55.89	−8.34	−0.51	−20.77	33.21	−58.41
12	−58.84	−14.34	−0.79	−20.27	32.05	−53.22
13	−66.11	−12.09	−0.63	−21.02	29.67	−60.54
14	−55.45	−11.38	−0.85	−19.06	31.39	−54.68
15	−60.33	−11.61	−0.90	−21.12	33.10	−58.92
16	−53.10	−10.15	−0.83	−19.14	30.54	−51.05
17	−57.23	−9.13	−0.60	−21.37	30.52	−56.32
18	−55.61	−7.26	−0.73	−19.64	29.52	−56.23
19	−56.20	−7.69	−0.20	−20.50	32.71	−59.02
20	−49.41	−7.47	−0.75	−17.90	33.04	−56.54
21	−56.15	−4.77	−0.75	−18.98	29.98	−61.53
22	−52.73	−3.09	−0.72	−19.60	31.87	−60.36
23	−52.68	−2.10	−0.51	−18.97	29.20	−59.59
24	−48.05	−12.37	−0.94	−17.01	38.45	−56.47
25	−48.42	−5.05	−0.77	−18.65	30.98	−55.36
26	−46.32	−10.32	−1.10	−15.22	33.57	−53.33
27	−45.10	−5.90	−0.65	−16.41	34.05	−56.03
28	−50.59	−11.25	−0.45	−17.39	34.95	−57.27
29	−51.62	−7.41	−0.26	−17.69	30.53	−56.90
30	−47.57	−7.74	−0.83	−17.39	33.23	−55.93
31	−50.34	−8.13	−0.75	−18.29	34.60	−57.68
32	−47.78	−6.75	−0.78	−15.91	31.79	−55.79
33	−45.48	−4.84	−0.72	−16.92	32.74	−55.57
34	−49.16	−8.09	−0.65	−16.95	32.36	−55.86
35	−51.47	−8.23	−0.78	−16.56	30.38	−54.85

36	−48.62	−7.25	−0.76	−17.40	32.30	−54.36
37	−50.38	−12.08	−0.69	−17.72	39.14	−57.94
38	−42.58	−5.93	−0.74	−15.76	34.50	−53.50
39	−47.62	−13.58	−0.82	−15.54	38.34	−56.78
40	−51.61	−8.59	−0.75	−17.92	35.50	−58.44
41	−55.22	−6.37	−0.69	−18.08	29.08	−58.82
42	−52.17	−10.38	−0.76	−18.77	35.43	−57.10
43	−47.80	−5.92	−0.12	−16.75	28.31	−55.30
44	−52.08	−8.36	−0.72	−17.88	31.30	−55.27
45	−51.76	−7.53	−0.06	−18.79	34.20	−59.34
46	−59.65	−12.17	−0.23	−18.71	31.66	−61.15
47	−53.96	−9.32	−0.17	−18.37	31.57	−58.19
48	−49.72	−7.68	−0.16	−17.17	32.90	−57.79
49	−53.00	−10.04	−0.15	−17.23	34.05	−61.18
50	−54.83	−9.71	−0.17	−17.46	31.35	−59.64
51	−54.37	−8.75	−0.07	−18.81	32.70	−60.36
52	−51.83	−6.67	−0.11	−19.26	36.24	−62.58
53	−50.89	−9.20	−0.10	−17.02	32.37	−58.90
54	−49.77	−8.71	−0.70	−17.16	32.57	−56.70
55	−51.71	−7.16	0.00	−17.53	30.34	−58.10
56	−49.25	−12.56	−0.11	−16.66	37.42	−57.36
57	−52.52	−10.24	−0.06	−17.03	33.97	−59.70
58	−62.78	−10.88	−0.04	−20.27	34.01	−65.86
59	−56.85	−13.31	−0.10	−19.46	38.02	−63.70
60	−54.55	−10.36	−0.10	−17.99	36.29	−62.76
61	−53.70	−8.89	−0.08	−20.57	37.93	−62.27
62	−59.54	−10.64	−0.04	−19.69	34.38	−64.19
63	−53.29	−10.48	−0.03	−19.56	37.94	−61.42
64	−50.92	−7.28	−0.22	−18.17	34.11	−59.06
65	−59.93	−10.77	−0.52	−19.89	33.39	−63.18
66	−53.08	−6.58	−0.69	−19.65	35.18	−60.94
67	−54.16	−7.32	−0.27	−18.82	33.92	−60.00
68	−54.94	−10.26	−0.25	−19.20	34.69	−58.00
69	−53.74	−11.03	−0.81	−18.35	35.97	−59.68
70	−52.43	−9.97	−0.60	−18.16	32.10	−55.85
71	−53.35	−8.52	−0.36	−18.41	33.84	−60.78
72	−52.24	−11.52	−0.59	−18.24	35.57	−56.99
73	−53.24	−11.12	−0.50	−17.44	34.65	−58.86
74	−52.17	−12.34	−0.35	−18.36	34.43	−54.91
75	−56.44	−12.34	−0.73	−19.82	33.77	−58.00
76	−55.92	−11.48	−0.75	−18.92	35.43	−59.82
77	−60.72	−11.53	−0.56	−20.60	33.25	−61.45
78	−58.02	−9.36	−0.26	−20.58	34.60	−62.83
79	−57.47	−11.29	−0.76	−20.35	37.34	−61.35
80	−51.29	−11.66	−0.13	−18.51	36.25	−58.74

81	−55.42	−11.68	−0.49	−18.83	33.98	−58.20
82	−51.71	−11.22	−0.73	−18.31	37.16	−58.95
83	−54.69	−13.38	−0.77	−16.94	34.52	−58.15
84	−60.45	−13.22	−0.63	−20.96	38.09	−63.00
85	−51.58	−8.67	−0.54	−19.07	36.29	−59.30
86	−56.42	−14.82	−0.84	−19.69	39.46	−61.71
87	−57.78	−14.12	−0.78	−19.71	39.34	−62.13
88	−56.26	−9.80	−0.74	−19.04	32.58	−60.01
89	−53.93	−12.21	−0.60	−19.52	37.59	−60.35
90	−55.80	−12.10	−0.20	−19.83	36.35	−59.56
91	−56.87	−12.87	−0.78	−20.08	35.99	−57.87
92	−60.95	−14.11	−0.71	−20.90	36.80	−62.82
93	−55.35	−12.82	−0.47	−18.14	36.52	−60.36
94	−59.14	−15.67	−0.73	−18.67	36.22	−60.30
95	−55.02	−13.21	−0.17	−19.59	36.01	−60.93
96	−49.28	−11.04	−0.67	−17.58	35.75	−57.79
97	−55.74	−9.09	−0.48	−20.36	36.15	−63.03
98	−53.12	−14.15	−0.66	−19.90	41.00	−62.30
99	−58.23	−8.75	−0.03	−20.04	32.96	−62.61
100	−54.16	−7.32	−0.27	−18.82	33.92	−60.00
**Maximum**	−66.11	−15.67	−1.10	−21.37	28.31	−65.86
**Minimum**	−42.58	−2.10	0.00	−15.22	41.00	−48.92
**Average**	−53.25	−9.72	−0.54	−18.56	34.23	−58.70
**STD (±)**	4.19	2.69	0.30	1.39	2.63	2.92

Table S2: MM-GBSA binding free energies components for the 2HCK-5d Complex obtained from molecular dynamics trajectories.

Frame No.	MMGBSA					
	ΔG Bind	ΔG Bind Coulomb	ΔG Bind H bond	ΔG Bind Lipo	ΔG Bind Solv GB	ΔG Bind VDW
0	−43.22	−13.06	−0.85	−13.97	24.97	−42.40
1	−33.74	−14.84	−1.92	−11.09	24.99	−31.17
2	−40.48	−7.02	−1.81	−14.28	22.90	−40.31
3	−40.92	−13.30	−1.90	−17.54	23.81	−36.28
4	−37.26	−2.47	−0.11	−16.82	24.01	−43.82
5	−37.97	−4.77	−0.09	−14.53	18.48	−38.76
6	−29.91	−9.57	−0.67	−13.19	27.06	−35.13
7	−31.02	−2.61	−0.01	−12.87	21.08	−36.71
8	−33.34	−7.15	−0.02	−12.11	21.60	−36.37
9	−40.36	−6.61	−0.04	−15.13	21.34	−41.14
10	−44.83	−6.54	−0.16	−15.53	22.14	−45.08
11	−38.79	−5.82	−0.01	−14.54	22.23	−41.12
12	−37.17	−7.42	0.00	−13.70	20.61	−37.06
13	−39.72	−8.84	−0.03	−13.57	23.19	−40.82
14	−40.14	−9.26	−0.02	−14.67	23.07	−40.38
15	−40.25	−7.76	−0.03	−14.92	23.03	−42.15
16	−42.99	−1.65	−0.04	−15.72	19.90	−46.01
17	−34.67	−7.15	0.00	−13.86	24.35	−39.39
18	−39.82	−5.34	−0.06	−14.30	20.18	−40.65
19	−35.37	−11.06	−0.07	−13.45	23.45	−34.65
20	−41.40	−5.67	−0.01	−14.79	21.86	−42.91
21	−37.15	−9.65	0.00	−13.01	22.63	−36.20
22	−37.30	−11.87	0.00	−13.53	24.28	−36.43
23	−33.18	−7.84	0.00	−13.42	25.54	−37.61
24	−37.94	−6.27	−0.06	−14.05	22.89	−41.17
25	−35.21	−7.87	0.00	−13.24	21.46	−36.21
26	−36.16	−7.74	0.00	−13.08	20.79	−36.31
27	−35.50	−8.00	0.00	−12.74	21.66	−36.44
28	−41.33	−7.12	−0.02	−14.86	23.92	−43.64
29	−39.35	−10.03	0.00	−15.17	25.20	−40.17
30	−37.55	−7.36	−0.01	−14.96	24.37	−41.27
31	−35.71	−7.43	−0.02	−13.80	25.11	−40.18
32	−36.34	−9.37	−0.02	−13.83	23.83	−38.79
33	−35.21	−10.20	0.00	−12.67	23.40	−35.49
34	−38.89	−7.18	−0.05	−14.01	23.72	−41.80
35	−34.60	−9.61	−0.01	−13.10	24.30	−36.83
36	−28.67	−5.82	0.00	−13.09	26.09	−38.03
37	−34.50	−8.93	0.00	−13.54	24.94	−36.96
38	−41.61	−6.62	−0.05	−14.35	23.37	−44.44

39	−35.30	−7.86	−0.04	−12.59	22.36	−37.87
40	−35.43	−7.09	−0.02	−14.16	21.69	−36.47
41	−42.24	−4.39	−0.26	−15.39	21.37	−44.33
42	−38.38	−5.60	−0.08	−14.54	23.08	−41.51
43	−40.19	−1.85	−0.09	−14.52	20.90	−45.13
44	−35.90	−7.79	−0.02	−13.37	25.11	−41.02
45	−44.39	−6.88	−0.40	−14.80	20.73	−43.89
46	−52.98	−8.90	−0.56	−17.21	21.55	−48.77
47	−42.35	−3.61	−0.21	−15.30	22.28	−45.78
48	−50.57	−8.82	−0.53	−16.93	23.06	−47.75
49	−34.19	−7.16	0.00	−12.06	20.84	−35.96
50	−39.40	−6.25	−0.10	−14.64	23.97	−42.80
51	−39.73	−7.72	−0.01	−15.04	20.93	−37.95
52	−39.05	−7.17	−0.01	−14.03	21.18	−38.90
53	−31.76	−6.93	0.00	−11.91	21.79	−34.48
54	−38.32	−6.72	0.00	−12.89	21.18	−40.16
55	−38.15	−7.27	−0.01	−14.64	22.48	−38.89
56	−39.07	−5.50	−0.01	−14.44	19.84	−39.21
57	−39.72	−7.36	−0.14	−14.67	21.24	−38.45
58	−40.32	−6.00	0.00	−15.54	19.73	−38.53
59	−39.03	−4.38	0.00	−15.96	21.57	−40.74
60	−35.86	−3.33	−0.01	−14.61	19.73	−38.01
61	−36.86	−8.09	−0.01	−12.63	22.10	−38.38
62	−35.95	−8.39	−0.04	−12.94	22.08	−36.41
63	−33.58	−7.91	0.00	−12.90	21.22	−33.89
64	−38.27	−7.63	0.00	−13.65	21.97	−39.37
65	−39.13	−6.95	−0.02	−11.29	17.72	−39.14
66	−24.68	−5.40	−0.25	−8.26	15.66	−27.21
67	−27.38	−4.37	−0.04	−10.39	16.57	−30.40
68	−23.05	−1.85	−0.16	−7.45	13.04	−26.29
69	−19.27	−3.72	0.00	−7.62	11.50	−19.49
70	−20.54	−0.90	0.00	−7.99	11.31	−25.59
71	−19.47	−4.19	0.00	−5.31	8.72	−21.67
72	−22.43	−8.70	−0.02	−6.88	13.58	−23.76
73	−16.86	−0.35	0.00	−5.63	8.37	−19.06
74	−44.52	−1.34	−0.30	−23.08	18.43	−41.25
75	−17.36	−1.80	0.00	−4.62	7.89	−19.13
76	−15.83	−3.29	−0.02	−6.55	10.20	−18.86
77	−17.46	−2.94	0.00	−6.37	11.29	−19.37
78	−34.13	−8.02	−0.64	−16.10	14.70	−24.93
79	−33.14	−3.28	−0.05	−14.42	10.32	−26.89
80	−34.14	−5.45	−0.02	−15.53	11.34	−23.39
81	−32.19	−5.18	0.00	−13.81	12.10	−26.64
82	−39.75	−8.01	−1.19	−15.25	17.32	−31.98
83	−26.85	−2.95	0.00	−11.24	11.12	−25.27

84	−25.43	−4.10	−0.61	−12.68	15.34	−24.08
85	−22.84	−7.69	−0.30	−5.36	10.92	−21.37
86	−20.93	−8.05	−0.63	−4.30	9.33	−20.28
87	−23.79	−11.11	−1.53	−9.02	18.11	−21.93
88	−28.65	−2.14	0.00	−12.64	11.37	−25.16
89	−31.44	−5.29	−0.18	−15.19	16.99	−26.16
90	−35.28	−5.37	−0.44	−15.12	17.10	−32.10
91	−36.77	−9.14	−0.80	−14.24	18.66	−30.66
92	−22.44	−6.22	−0.22	−5.53	14.79	−25.65
93	−31.30	0.21	−0.16	−14.15	13.38	−29.57
94	−26.56	−3.20	−0.34	−11.99	17.32	−29.32
95	−30.50	−10.89	−0.59	−8.97	20.54	−32.02
96	−24.90	−0.87	−0.01	−11.89	7.56	−18.91
97	−27.81	−0.42	0.00	−13.63	11.18	−23.91
98	−24.91	−0.68	−0.03	−13.60	12.53	−22.52
99	−22.37	−1.50	0.00	−9.04	9.66	−21.68
100	−28.65	−2.14	0.00	−12.64	11.37	−25.16
**Maximum**	−52.98	−14.84	−1.92	−23.08	7.56	−48.77
**Minimum**	−15.83	0.21	0.00	−4.30	27.06	−18.86
**Average**	−34.15	−6.28	−0.19	−12.93	19.24	−34.61
**STD (±)**	7.53	3.06	0.40	3.15	5.13	7.98

Table S3: MM-GBSA binding free energies components for the 2QV4-5d Complex obtained from molecular dynamics trajectories.

Frame No.	MMGBSA					
	ΔG Bind	ΔG Bind Coulomb	ΔG Bind H bond	ΔG Bind Lipo	ΔG Bind Solv GB	ΔG Bind VDW
0	−58.80	−13.22	−1.33	−24.03	23.68	−41.83
1	−39.42	−5.75	−0.06	−18.61	17.74	−30.82
2	−58.32	−10.30	−1.03	−23.40	17.88	−43.08
3	−60.39	−2.17	−0.47	−25.95	16.29	−46.10
4	−60.28	−3.37	−0.72	−25.52	17.85	−47.72
5	−56.12	−8.97	−0.62	−25.22	22.31	−46.31
6	−59.56	−2.14	−0.66	−24.75	14.73	−45.33
7	−63.86	−11.27	−1.04	−25.26	17.72	−47.60
8	−58.33	−2.22	−0.37	−24.92	15.94	−44.70
9	−61.53	−7.79	−0.94	−25.39	17.09	−43.38
10	−55.05	−4.75	−0.49	−25.01	17.13	−41.66
11	−61.62	−7.68	−0.86	−24.20	17.02	−44.75
12	−58.94	−3.19	−0.61	−24.59	16.45	−44.76
13	−67.66	−7.11	−0.80	−26.22	16.06	−48.61
14	−61.29	−7.58	−0.49	−26.43	20.51	−50.47
15	−58.58	−5.10	−0.53	−25.68	17.57	−47.19
16	−55.00	−6.02	−0.05	−25.47	22.46	−43.54
17	−62.42	−6.43	−0.68	−26.61	18.04	−47.42
18	−64.44	−4.06	−0.59	−26.49	16.87	−49.13
19	−62.66	−3.72	−0.48	−26.95	17.12	−50.92
20	−60.40	−3.42	−0.69	−25.27	19.08	−49.26
21	−63.66	−5.39	−0.50	−26.15	16.06	−45.62
22	−67.41	−4.28	−0.47	−26.20	16.86	−50.25
23	−58.78	−6.16	−0.73	−24.48	20.43	−47.30
24	−59.79	−5.69	−0.58	−25.17	18.59	−46.11
25	−61.47	−3.49	−0.30	−26.41	18.94	−47.84
26	−63.27	−7.98	−0.60	−25.21	18.92	−49.19
27	−47.83	−5.63	−0.02	−19.98	15.84	−37.75
28	−29.11	1.32	0.00	−16.85	19.70	−33.21
29	−45.31	−1.90	−0.06	−21.18	20.04	−41.99
30	−48.33	−4.26	−0.54	−19.55	16.06	−39.67
31	−42.98	−7.04	0.00	−19.01	18.35	−34.66
32	−37.67	−6.32	0.00	−17.75	19.64	−32.31
33	−49.42	−6.57	−0.02	−19.59	17.63	−39.08
34	−46.63	−4.13	−0.07	−20.23	17.95	−40.07
35	−53.89	−1.62	−0.05	−23.56	19.87	−44.83
36	−46.48	−3.64	−0.17	−19.32	16.40	−39.97

37	−46.14	−6.41	−0.09	−18.90	17.88	−38.92
38	−46.42	−2.51	−0.04	−20.67	19.48	−43.06
39	−47.75	−6.97	−0.41	−20.22	18.33	−41.11
40	−48.37	−5.07	−0.42	−19.07	16.75	−40.70
41	−48.22	−3.10	−0.13	−20.73	19.62	−41.58
42	−50.75	−5.45	−0.46	−18.93	15.82	−42.20
43	−47.00	−5.34	−0.05	−18.91	18.44	−39.58
44	−54.90	−3.15	−0.25	−25.29	19.45	−43.28
45	−46.25	−6.64	−0.11	−18.22	17.71	−39.15
46	−49.25	−2.87	−0.24	−20.02	16.42	−40.01
47	−51.99	−5.06	−0.30	−18.86	17.89	−43.96
48	−51.33	−3.64	−0.31	−19.72	16.49	−42.75
49	−46.37	−3.22	−0.07	−19.50	18.62	−41.01
50	−56.95	−0.36	−0.34	−24.68	16.24	−44.39
51	−57.57	−2.29	−0.14	−21.84	14.13	−43.56
52	−47.17	−6.17	0.00	−21.53	21.12	−37.23
53	−45.16	−1.57	0.00	−19.53	15.92	−37.25
54	−44.88	−4.36	0.00	−21.40	20.22	−36.26
55	−38.73	2.89	0.00	−19.70	14.78	−34.82
56	−41.68	−4.33	0.00	−17.45	15.07	−33.57
57	−31.29	0.59	0.00	−17.80	12.76	−27.64
58	−29.03	2.89	−0.01	−15.16	12.00	−28.82
59	−36.94	−0.30	−0.59	−18.91	17.09	−34.08
60	−39.82	−1.75	−0.61	−19.23	17.89	−35.91
61	−34.78	−1.84	−0.61	−17.83	16.93	−32.20
62	−33.75	3.19	−0.04	−17.15	11.71	−32.84
63	−31.88	1.59	0.00	−16.92	13.13	−29.98
64	−45.27	−8.43	−0.53	−18.42	14.75	−32.40
65	−37.17	1.54	0.00	−19.52	13.46	−32.36
66	−41.08	−7.31	−0.53	−17.39	14.97	−31.03
67	−43.77	−10.69	−0.56	−19.54	18.42	−31.77
68	−42.81	−8.43	−0.55	−17.33	14.91	−31.62
69	−38.05	−5.16	−0.58	−18.36	17.07	−31.63
70	−40.73	−4.75	−0.54	−17.97	14.01	−30.87
71	−37.75	2.89	0.00	−18.74	12.56	−34.67
72	−34.59	0.93	−0.04	−17.64	17.04	−34.78
73	−33.65	2.55	0.00	−17.57	12.33	−31.47
74	−42.56	−7.72	−0.52	−17.53	13.10	−30.69
75	−44.92	−2.86	−0.52	−20.34	14.31	−34.48
76	−39.21	−7.40	−0.49	−17.38	16.90	−31.26
77	−34.67	0.55	−0.04	−18.43	14.77	−33.54
78	−42.54	−6.87	−0.53	−18.60	17.90	−34.57
79	−32.60	1.01	−0.02	−17.19	16.43	−33.56
80	−34.66	2.13	−0.02	−16.77	14.15	−34.00
81	−29.98	2.11	0.00	−14.53	11.54	−28.63

82	−32.05	3.04	−0.02	−16.22	11.50	−29.87
83	−32.32	2.22	−0.01	−15.64	12.50	−30.85
84	−34.45	2.43	−0.01	−17.74	13.26	−32.34
85	−38.88	−7.32	−0.56	−18.03	17.83	−30.89
86	−42.59	−7.90	−0.54	−18.33	17.27	−33.75
87	−38.74	−8.59	−0.53	−17.93	17.00	−29.02
88	−42.79	−7.14	−0.53	−18.64	14.68	−31.48
89	−41.07	−9.85	−0.55	−18.00	17.51	−30.74
90	−39.96	−3.41	−0.43	−19.44	15.85	−32.38
91	−39.69	−6.91	−0.52	−18.86	15.79	−30.73
92	−32.81	−0.87	−0.22	−17.98	15.51	−31.88
93	−31.07	2.28	−0.35	−17.32	21.46	−37.15
94	−30.91	−0.62	−0.07	−17.01	14.68	−29.32
95	−29.92	1.33	0.00	−14.26	12.44	−29.61
96	−35.21	−1.84	−0.25	−16.81	16.38	−32.79
97	−32.40	2.03	0.00	−16.81	13.58	−32.02
98	−30.68	3.12	0.00	−16.86	13.33	−29.78
99	−31.04	2.82	−0.01	−15.29	8.62	−27.58
100	−34.45	2.43	−0.01	−17.74	13.26	−32.34
**Maximum**	−67.66	−13.22	−1.33	−26.95	8.62	−50.92
**Minimum**	−29.03	3.19	0.00	−14.26	23.68	−27.58
**Average**	−45.92	−3.55	−0.33	−20.37	16.57	−37.90
**STD (±)**	10.89	3.91	0.30	3.46	2.67	6.65
